# Water Electrolysis Beyond Platinum: Carbon Nitride Materials for Electrochemical Hydrogen Evolution

**DOI:** 10.1002/advs.202514030

**Published:** 2025-09-23

**Authors:** Kamel Eid, Markus Antonietti

**Affiliations:** ^1^ Department of Colloid Chemistry Max Planck Institute of Colloids and Interfaces Research Campus Golm D‐14424 Potsdam‐Golm Germany; ^2^ Gas Processing Center (GPC) at the College of Engineering Qatar University 2713 Doha Qatar

**Keywords:** electrocatalysis, green hydrogen, hydrogen storage, pt‐replacement, water splitting

## Abstract

A facile approach is presented for the scalable generation of porous, ultralong carbon nitride nanoscrolls as electrocatalysts for electrochemical hydrogen storage and evolution. Activation of C_3_N_4_ by subpotential reduction reveals a new material that stores and finally evolves hydrogen, with a performance being superior to Platinum. The optimized system displays an overpotential (η_10_ = 7 mV) close to commercial Pt/C but provides a superior current density (up to ≈1.3 A cm^−2^), turnover frequency, HER rate, and higher stability. Additionally, at underpotential conditions, ≈1.5 wt.% activated hydrogen can be stored at ambient pressure and room temperature in such materials.

## Graphical abstract

Porous long carbon nitride nanoscrolls can be activated by subpotential reduction to become effective electrocatalyts for electrochemical hydrogen evolution (HER) and storage, with a performance superior to Platinum. The optimized system displays an overpotential close to commercial Pt/C but provides a superior current density (1.3 A/cm2), turnover frequency, HER rate, and higher stability.

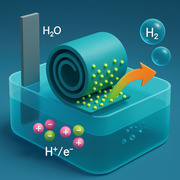

## 1 Introduction

The exploration of alternative electrocatalysts replacing expensive and rare Pt‐group metals is a critical prerequisite to install a global energy system based on low‐cost hydrogen evolution reaction (HER).^[^ ^1^ ^]^ This drove the development of various “affordable” catalysts, including some based on more common transition metals (also as carbides, phosphides, chalgonides)^[^ ^2^ ^]^ and carbon‐based covalent materials.^[^ ^3^ ^]^ In most of those cases, stability and performance are still far from allowing possible applications in a real‐life device. Carbon Nitride nanostructures, on the other hand, show an appealing electronic band structure, simple synthesis from earth‐abundant educts, outstanding physicochemical durability, sufficient surface area, possibility to craft channels and pores, and electron and ion transport.^[^ ^4^ ^]^ When doped with metal nanoparticles and single‐metal atoms, the first publications indicate their suitability for HER.^[^ ^5^ ^]^ However, their overall current density (≤100 mA cm^−2^), overpotential (down to 71 mV), as well as their instability are still far below the gold standard of noble metals.^[^ ^6^ ^]^

g‐C_3_N_4_ deposited on carbon carriers was predicted theoretically to be close to the optimal point of the vulcano plot for hydrogen evolution,^[^ ^7^ ^]^ but also here, performance is limited by the lower electrical conductivity of the semiconducting carbon nitride. Electron transport was addressed by creating nanocomposites with highly conductive N‐graphene^[^ ^8^ ^]^ or with metals/metal sulfides, such as in Au‐aerogel‐CN*x*,^[^ ^9^ ^]^ MoS_2_/g‐C_3_N_4_,^[^ ^10^ ^]^ and promising activities with moderate overpotentials (≈140–185 mV) were described.^[^ ^11^ ^]^ Metal doping as single atoms into the skeleton structure of g‐C_3_N_4_ also changes electronic properties while adding catalytically active sites.^[^ ^6a^ ^]^ This includes Cu‐doped g‐C_3_N_4_ nanosheets,^[^ ^12^ ^]^ Ir single atom/g‐C_3_N_4_ nanosheets (Ir‐g‐CN),^[^ ^13^ ^]^ Cu‐doped g‐C_3_N_4_ nanofibers,^[^ ^14^ ^]^ and MnFeCo^[^ ^15^ ^]^ doped g‐C_3_N_4_ nanofibers. All these reports describe performances still far below the Pt reference (Table S1, Supporting Information).

Stimulated by on/off record high hydrogen uptakes of carbon nitrides at 77 K in the lab, we resolved the reproducibility issue and describe in this paper an approach to load hydrogen electrochemically into a carbon nitride‐hydrogen nanohybrid. Electron‐proton couples are intercalated into ion‐doped g‐C_3_N_4_, and the hydrogen loading activates the electrocatalyst to show a low impedance and improved HER activity.

The synthesis starts with a previously optimized, rational, and scalable fabrication of g‐C_3_N_4_ nanoscrolls. For synthesizing them as 2d layers which subsequently scroll up, we atomically codope the layers with Mn or mixed metal (M) (M = Co, Cu, and Fe) ions (MnM/g‐C_3_N_4_). At low metal ion doping levels (≈1.6±0.4 wt.%), we obtain porous, ultra‐long, scrolled nanostructures (5–12 µm in length and 70–90 nm in width) with a comparably high surface area (140–308 m^2^ g^−1^, high for carbon nitrides). It should be already stated here that the electrochemical performance is not directly related to the ions, but that ions are needed to form the nanostructure. With the scrolling, activation of the carbon nitrides is promoted, and it is the rolled single carbon nitride sheet which could be easily activated by electrochemical proton‐electron transfer (PET).

The electrochemical HER performance of MnM/g‐C_3_N_4_ with and without reductive‐activation is then benchmarked against pristine g‐C_3_N_4_ and commercial Pt/C (10 wt.% Pt). The as‐made electron‐proton intercalation compound of carbon nitride exhibits an impedance behavior better than the Pt‐reference and finally offers an electrocatalytic HER activity superior to all previous Pt‐replacement materials (Table S1, Supporting Information).

## 2 Results and Discussion

The special g‐C_3_N_4_ nanostructure was created from melamine monomers protonated with HNO_3_ in an aqueous solution of methanol under stirring, adding minor amounts of metal precursors when needed.^[^ ^14^, ^15^ ^]^ Melamine is easily polymerized into melem after protonation of NH_2_ by H while eliminating NH_3,_ which also neutralizes parts of the primary acid, and the polymerization process to melem can be accomplished at comparably low temperatures (≤200 °C). Melone and more highly condensed g‐C_3_N_4_ are obtained by further heating of the collected precipitates at elevated temperatures (≥500 °C).^[^ ^16^ ^]^ Metal atoms are directly integrated during the polycondensation process, presumably as counterions of imide anions located between the melem subunits. The as‐formed thin 2D carbon nitride sheets long long‐range conjugated and thereby strongly polarizable and van der Waals attractive. This is why they tend to roll up to reduce their primary high surface‐free energy, whereas continued simultaneous release of ammonia and ammonium salts forms abundant macropores.^[^ ^15^ ^]^ The model of a detailed formation mechanism with supporting analyses is shown in Figures S1 and S2, (Supporting Information).

**Figure 1:**
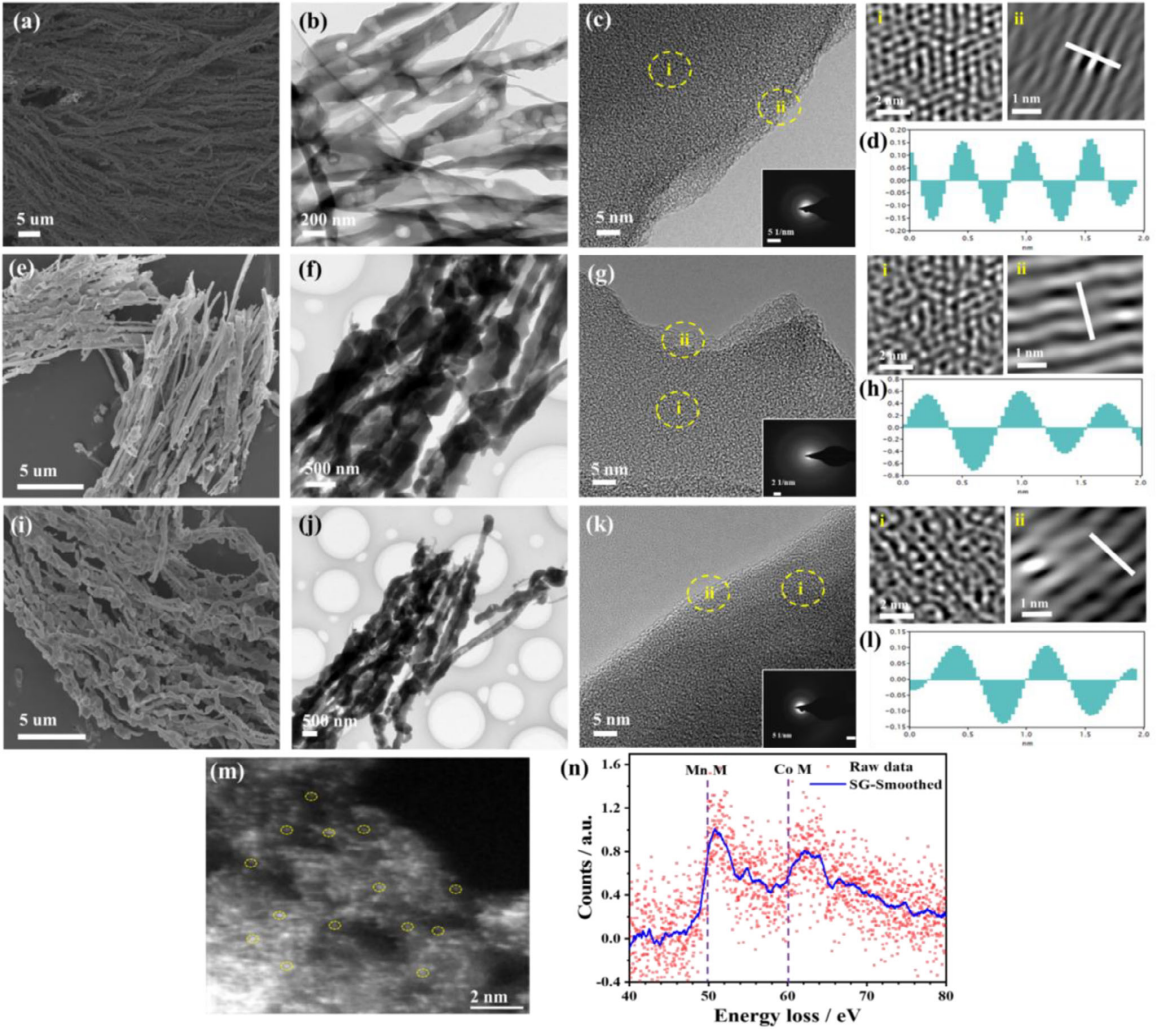
SEM images (a, e, and i), TEM images (b, f, and j), HRTEM images (c, g, and k), and FFT Fourier transform filtering (FFT) images of the marked areas in HRTEM images with their related histogram of lattice spacing (d, h, and l) of MnCo/g‐C_3_N_4_, MnCu/g‐C_3_N_4_, and MnFe/g‐C_3_N_4_, respectively. The insets in (c, g, and k) are the relevant SAED. The spherical aberration‐corrected TEM image (m) and electron energy‐loss spectrum (EELS) of MnCo/g‐C_3_N_4_ (n). Note that the EELS spectra are made on a small number of single metal atoms.

The SEM images show the ultra‐long fibers of MnCo/g‐C_3_N_4_ (**Figure** **1**a)_,_ MnCu/g‐C_3_N_4_ (Figure 1e), and MnFe/g‐C_3_N_4_ (Figure 1i) with average length/width of 12 µm/70±5 nm, 7.8 µm/85±6 nm, and 5 µm/90±6 nm, respectively. The TEM images verify the uniformity of the porous necklace‐like features of MnM/g‐C_3_N_4_ (Figure 1b,f,j). The shown sample discloses a rough surface, multiple twists, surface steps, and a high hierarchical porosity. The hierarchical porosity is beneficial for accelerating the transport to possible active sites. HRTEM images of the different MnM/g‐C_3_N_4_ confirm their uniformity with a view onto the planes in the middle of the structures and a view on the perpendicular graphitic stacking in the outer zones, as it is typical for TEM of scrolled 2D structures (Figure 1c,g,k).

Notably, we could not find any regular structures of metal nanoparticles, which supports the expected distribution as single counterions, as it is typical for single metal‐atom‐doped g‐C_3_N_4_ nanostructures.^[^ ^12^, ^14^, ^15^, ^17^ ^]^ The regularly spaced density lines in the outer parts of the scrolls exhibit an adjacent graphitic interlayer spacing of 0.35–0.38 nm, assigned to the (002) stacking of the g‐C_3_N_4_ structure. This is slightly expanded when compared to the crystalline systems with translational order (d = 0.32 nm), but the expansion is a typical feature of scrolls and tubes that have to handle curvature in their packing. It is also to be mentioned that such an expansion highly promotes cation transport through the structure, as we know from battery carbon electrodes. The selected area electron diffraction (SAED) demonstrates the polycrystalline nature of the scrolls (insets in Figure 1e,h,m). To gain more profound insights into the regularity of the synthesized materials, we conducted a Fourier transform filtering (FFT) analysis on the HRTEM images and their related histogram of lattice spacing of the samples (Figure 1d,h,l). The analysis reveals that the outer layer consists of graphite domains characterized by multiple well‐aligned layers, significant curvatures, and various bends and crystalline defects. The FFT images of the inner regions of the skolls reflect an atypical top‐on view onto layers with a honeycomb‐like arrangement of carbon and nitrogen atoms, accompanied by various layer‐over‐layer interference artifacts. The spacing between adjacent graphite planes ranges from 0.3 to 0.45 nm, which is slightly larger than the d‐spacing of the (002) crystal plane of graphite carbon nitride. This is as discussed above typical foll rolled nanolayer structures.

The presence of metal single atoms was confirmed by spherical aberration‐corrected TEM analysis, which demonstrates a homogeneous distribution of atoms, smeared out by their high thermal mobility onto the soft 2D‐sheets with an average diameter of 0.1–0.12 nm (Figure 1m). Through the experiments, one can literally observe the atoms moving as a consequence of the high flux of electrons. Making the spots about twice as big as observed on more rigid supports.

The EELS was recorded across the M_2,3_ region, revealing two distinct M‐edge features: the Mn M2,3 at 50–53 eV and the Co M_2,3_ feature at 62–66 eV (Figure 1n). This finding suggests the co‐existence of the MnCo single atom. The spectra are very noisy due to the very low concentrations of atoms involved, but the peak shift from the standard position indicates high‐spin MnCo single‐atom sites coordinated with nitrogen, as further evidenced by the sharp M_3_ edge and a modest asymmetry between M_3_ and M_2_. The near‐unity M_3_/M_2_ ratio suggests substantial 3d unoccupied orbitals, consistent with the oxidation state.

**Figure 2:**
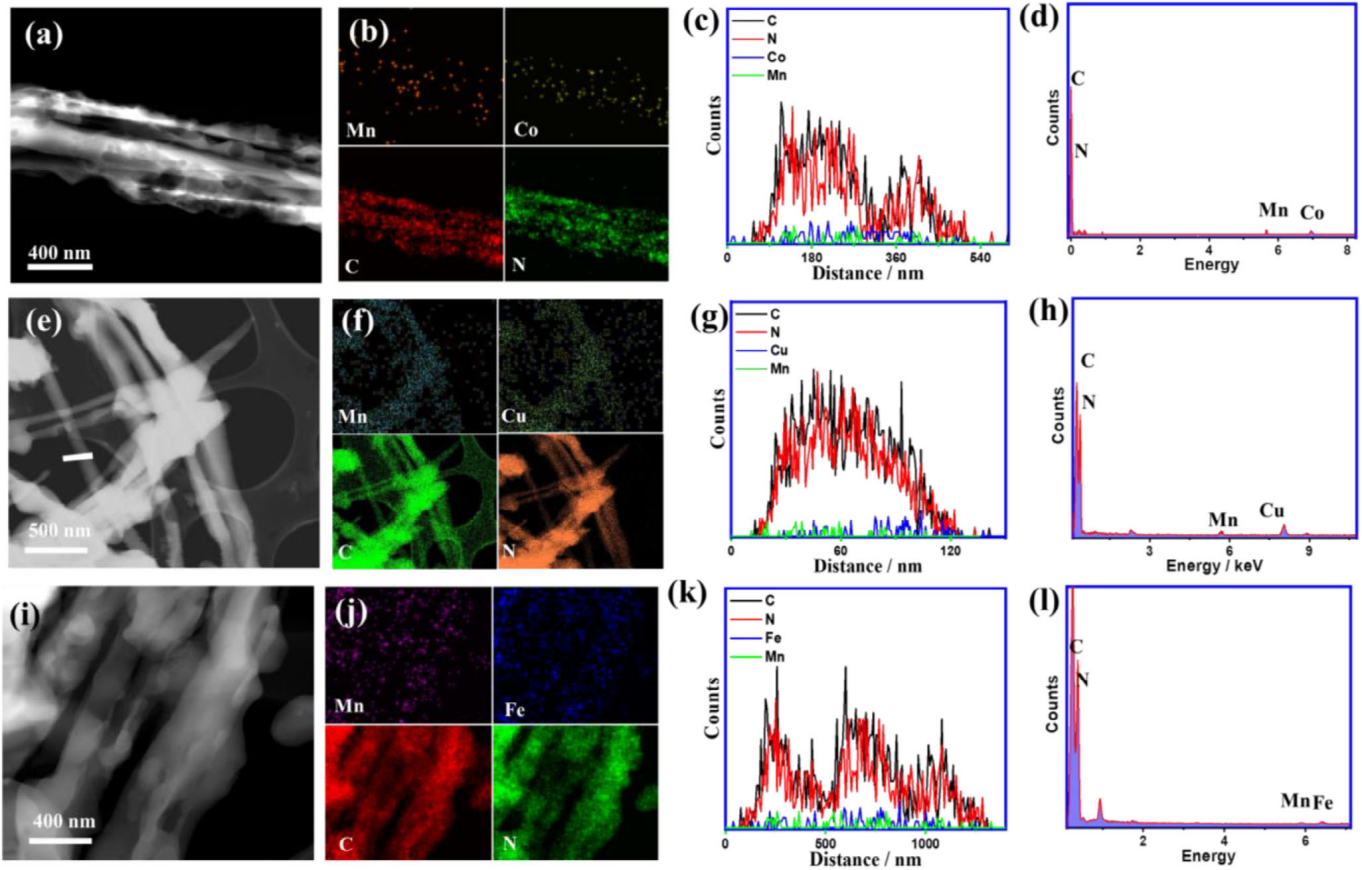
HAADF‐STEM images (a, e, and i), elemental mapping images (b, f, and j), EDS‐scan line profile (c, g, and k), and the EDX, respectively (d, h, and l) of MnCo/g‐C_3_N_4_, MnCu/g‐C_3_N_4_, and MnFe/g‐C_3_N_4_, correspondingly.

The HAADF‐STEM images again display the structural details of MnCo/g‐C_3_N_4_ (**Figure** **2**a), MnCu/g‐C_3_N_4_ (Figure 2e), and MnFe/g‐C_3_N_4_ (Figure 2i), including outer rough and twisted surfaces and porosity. MnCo/g‐C_3_N_4_ displayed an even higher porosity and more twisted texture, i.e., the presence of the different metal ions is indeed decisive for finer morphological differences. The elemental mapping, EDS‐scan line profile, and EDX validate the uniform distribution of both metals (≈1.6–1.7 wt.%) besides C and N in the skeleton structure of MnM/g‐C_3_N_4_ (Figure 2d,b,f,h,j,l; Table S2, Supporting Information). Mn/g‐C_3_N_4_ and metal‐free g‐C_3_N_4_ were also made by our synthetic technique and possess a rolled‐fiber‐like structure, but with less porosity relative to MnM/g‐C_3_N_4_ (Figure S3a,b, Supporting Information).

The diverse C_3_N_4_ fibers were also analyzed with XRD. A broad diffraction peak at a 2*θ* angle of 26.6° is ascribed to the graphitic interlayer stacking of the conjugated aromatic g‐C_3_N_4_ sheets^[^ ^18^ ^]^ (Figure S4a, Supporting Information). The peaks of MnM/g‐C_3_N_4_ fibers are broadened relative to Mn/g‐C_3_N_4_ and g‐C_3_N_4_ because smaller scrolls possess less long‐range translational order of the stacking. The absence of XRD diffraction peaks for pure metal dopants or their oxides (i.e., Cu, Mn, and Fe) is taken as a further indication of a single atom dispersion at low loading amounts in the inner vacancies of g‐C_3_N_4_, in line with many similar reports.^[^ ^12^, ^14^, ^15^, ^17^ ^]^ The effect of metal single‐atoms on the full peak width at half maximum (FWHM), crystallite size, microstrain, and d‐spacing of g‐C_3_N_4_ are summarized in (Table S3, Supporting Information). The FTIR analysis reveals the absorption bands attributed to the breathing mode tris‐s‐triazine rings, but with a noticeable broadening in the N–H peak of MnM/g‐C_3_N_4_ compared to g‐C_3_N_4_, plausibly attributed to the ionic nitrogen‐metal coordination and the coupled change in the vibrations of the N‐atoms (Figure S4b, Supporting Information).^[^ ^12^, ^14^, ^15^, ^17^ ^]^ The core‐level of valence states and surface composition of MnM/g‐C_3_N_4_ were investigated by XPS analysis (Figures S5 and S6, Supporting Information). The metal signals are very noisy, due to the low overall concentration of metals and their presence as a single atom, but the existence of metal dopants with various valence states can at least be proven. The obtained elemental composition determined by the XPS, EDX, and ICP is summarized in (Table S2, Supporting Information). The N_2‐ads/des_ isotherms of porous MnM/g‐C_3_N_4_ reflect the characteristics of multimodal pore‐size distributions. BET surface areas are determined as 308, 280, and 140 m^2^ g^−1^ for MnCo/g‐C_3_N_4_, MnCu/g‐C_3_N_4_, and MnFe/g‐C_3_N_4_, respectively, and pore diameters of 4–30 nm and pore volumes of 0.44–0.3 cm^3^ g^−1^ are obtained (Figure S7, Supporting Information).

**Figure 3:**
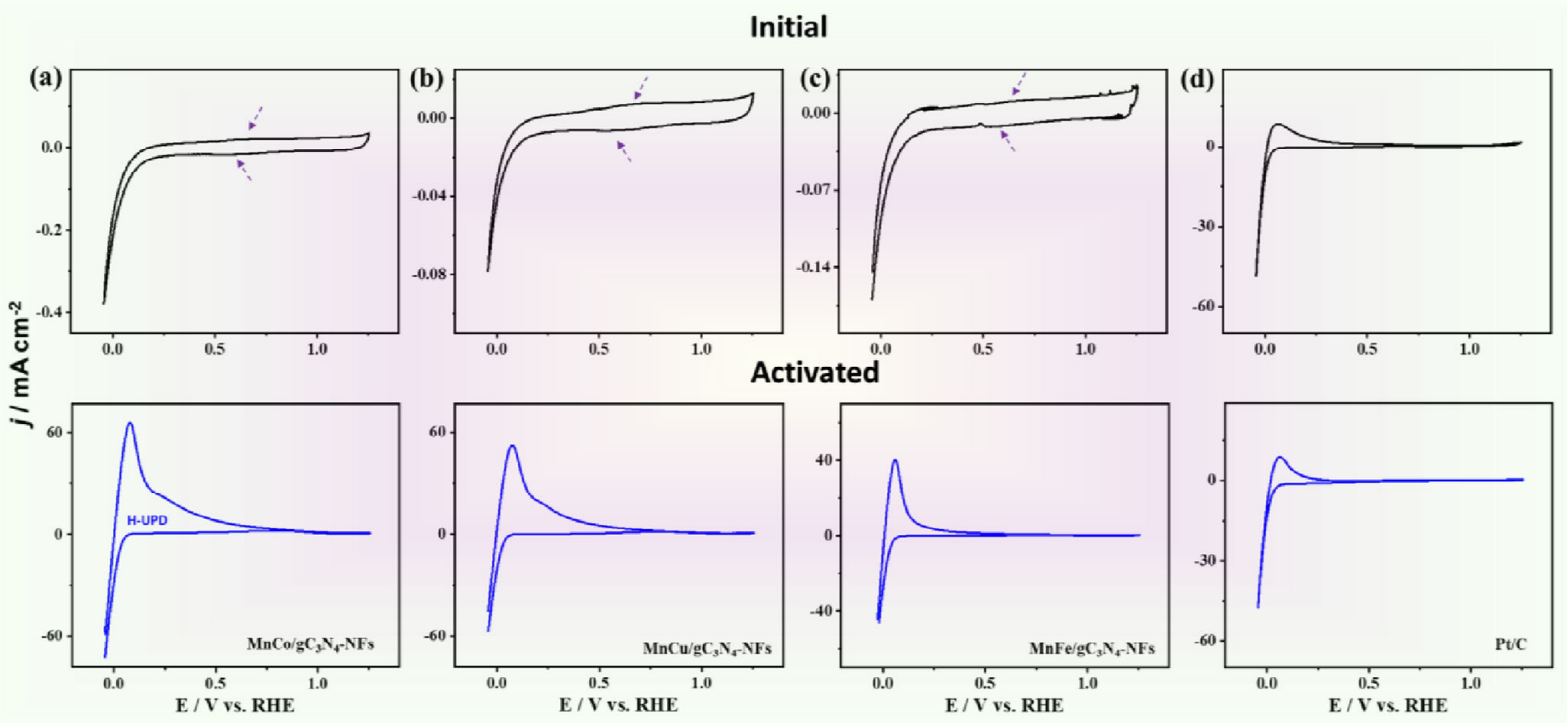
CV curves of activated and nonactivated electrocatalysts measured at 50 mV s^−1^. The arrows in a—d) refer to the formation of MnO and its reduction Mn‐H.

For the electrochemical experiments, the catalyst inks were coated on a carbon foam as working electrodes through volumetric casting (Supporting Information). The electrodes underwent reductive activation at an applied potential of −0.6 V for 3 h. However, all the electrocatalytic tests except CV were performed on the electrodes with stirring employed to remove any accumulated bubbles from the surface of the electrodes.^[^ ^19^ ^]^ The extent of the activation mechanism can be easily judged by a strongly increasing current well ahead of hydrogen evolution, which, however, saturates toward a faradaic redox peak. Once activated (and not reoxidized), the current response is immediate and stable (that is, the material as such has changed, i.e., activation comes with structural and composition changes) (**Figure** **3**). The cyclic voltammograms (CVs) curves of MnM/g‐C_3_N_4_ and Mn/g‐C_3_N_4_ measured in 0.5 M H_2_SO_4_ electrolyte reveal initially a quasi‐rectangular voltammogram in the 10 µA range attributed to a weak capacitive proton‐electron pair storage besides weak peaks assigned to the formation of M–H at high negative potentials and reduction of M–O at slightly positive potentials (Figure 3).^[^ ^14^, ^15^ ^]^ The relative extent of these events in relation to the reduction of the support, however, already clearly indicates that metal ions are not the main players in this electrochemical role play. Meanwhile, the reference Pt/C sample revealed the characteristic voltammogram of Pt and its hydrogen evolution. The electrochemically active surface areas (ECSAs) of MnM/g‐C_3_N_4_ were ≈29.7–19.6 cm^2^, and MnCo/g‐C_3_N_4_ showed the highest ESCA as calculated from measuring the CV at different scan rates (Figure S8, Supporting Information).^[^ ^15^, ^16^ ^]^

Notably, after the reductive activation of samples, the voltamogram of MnM/g‐C_3_N_4_ altered significantly and developed an impressive HER activity. This is mostly seen on the current scale, which is 4 orders of magnitude higher (i.e., now instead of 10 µA of the order of 100 mA). In the voltage region below the liberation of hydrogen, a strong subpotential Faradaic peak is recorded, which is analogous to the literature case of Pt, classically assigned to chemically bonded proton‐electron pairs or under‐potential adsorbed hydrogen (H‐UPD). Also, this peak is significantly larger than that one of Pt/C. The stored charges obtained from integration of H‐UPD peak area are Q_H_ = ≈31.67, 23.78, 8.82, and 5.22 mC on MnCo/g‐C_3_N_4_, MnCu/g‐C_3_N_4_, MnFe/g‐C_3_N_4_, and Pt/C, respectively. The storage is largely faradaic and comes with a coupled proton from the medium for every electron stored (MnM/g‐C_3_N_4_ + H^+^ + e^−^ → MnM/g‐C_3_N_4_‐H_ads_).

The observed electron storage in the diverse samples is massive, up to 6 times higher than Pt, and corresponds to the first‐in‐class, MnCo/g‐C_3_N_4_, to 1 H^+^/1 e^−^ pair per 60 mass units of the support, these are ≈3 electrons per heptazine pore, or 1.5 wt.% hydrogen stored at ambient pressure and temperature. The comparison with the platinum standard and the magnitude of the results suggest that proton‐electron pairs must be bound to g‐C_3_N_4_, as indeed the little metal present would be hopelessly overloaded with electrons and hydrogen. However, the metal single atoms can serve as active sites to enhance the HER performance, while their main role is to generate the scrolled nanostructure with expanded layer stackings, open transport channels, and a high specific surface area.

**Figure 4:**
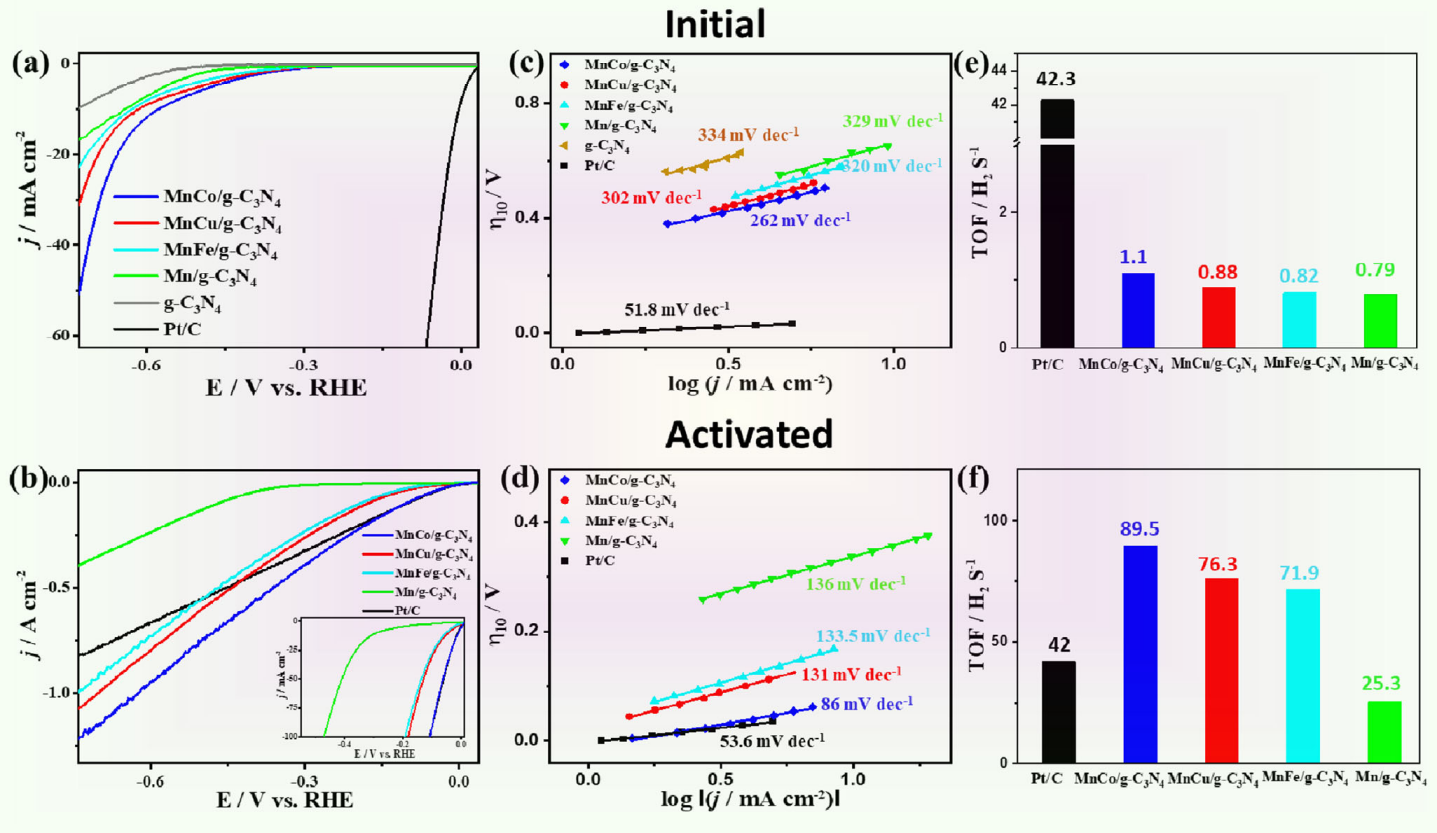
LSV at scan rate 2 mVs^−1^ a,b), Tafel plot (c,d), TOF at −0.6 V (d–f) of non‐activated, and activated MnM/g‐C_3_N_4_ and Pt/C, respectively.

In the initial HER experiment, g‐C_3_N_4_, both with and without MnM single atoms, exhibited inferior activity, with J_HER_ values ranging from 10 to 50 mA cm^−^^2^, but with a superior performance with MnM single atoms (**Figure** **4**a). The fact that MnCo/g‐C_3_N_4_ is in the unreduced state five times more effective than g‐C_3_N_4_ indicates the role of single atoms as active sites for HER, however at overpotentials very similar to other single and dual atom catalysts. Notably, the reductive activation significantly enhanced the performance of all catalysts, achieving J*_HER_* values between 0.25 and 1.3 A cm^−^^2^. However, MnM/g‐C_3_N_4_ catalysts outperformed g‐C_3_N_4_ by at least a factor of two, attributed to the influence of metal single‐atom active sites. MnCo/g‐C_3_N_4_ now unexpectedly outperformed the reference Pt/C catalyst (Figure 4b) in practically all aspects: at −0.73 V, the *J_HER_* of MnCo/g‐C_3_N_4_ reaches 1.3 A cm^−2^, compared to Pt/C (0.86 A cm^−2^) (Figure 4b; Table S5, Supporting Information). The onset potential is about the same as Pt/C, while the overpotential for 10 mA cm^−2^ (ƞ_HER_) of MnCo/g‐C_3_N_4_ is only 7 mV, compared to 577 mV before reductive activation of carbon nitride. As such, reduced MnCo/g‐C_3_N_4_ shows the highest reported HER activity among all Pt‐free electrocatalysts so far (Table S1, Supporting Information). After the activation, the Tafel slope of MnM/g‐C_3_N_4_ decreased significantly (i.e., from 262 to 86 mVdec^−1^ on MnCo/g‐C_3_N_4_), while commercial Pt/C stayed about constant (Figure 4c,d). Our slope implies a two‐electron HER process without impedance losses,^[^ ^14^, ^15^ ^]^ which we attribute to quick local chemical rearrangements of the stored proton‐electron pairs prior to H_2_‐dimerization. Then, hydrogen desorption becomes the rate‐limiting step. Notably, a higher Tafel slope at high currents points to mass transport limitations and the build‐up of impedance on the catalyst surface at those extreme currents, but the Tafel values of reduced g‐C_3_N_4_ are still lower than those of practically all other non‐Platinum electrocatalysts. Thereby, the reductive activation not only reduces overpotentials and Tafel slope, but also enables extremely high turnover frequencies (TOF), i.e., hydrogen liberation is both thermodynamically and kinetically accelerated. The TOF increased from 1.1 H_2_/atom sec on MnCo/ g‐C_3_N_4_ to 89.5 H_2_/atom sec, which is two times higher than that of Pt/C under the same applied bias potential (−0.6 V) (Figure 4e,f). This can be recalculated into a higher weight specific HER rate (4323 mol^.^g^−1.^h^−1^) on MnCo/g‐C_3_N_4_ (Table S5, Supporting Information). We take this as a reflection of a very high ion and electron conductivity of the reduced MnCo/g‐C_3_N_4_ support at the already mentioned high electron‐proton pair loadings of up to three H per structural pore. Recalculating the number of activated hydrogens back to the metal centers is unphysically and would exceed Pt by two orders of magnitude.^[^ ^14^, ^15^ ^]^ These results warrant the significant effect of MnM single atoms on accelerating the activation process.

**Figure 5:**
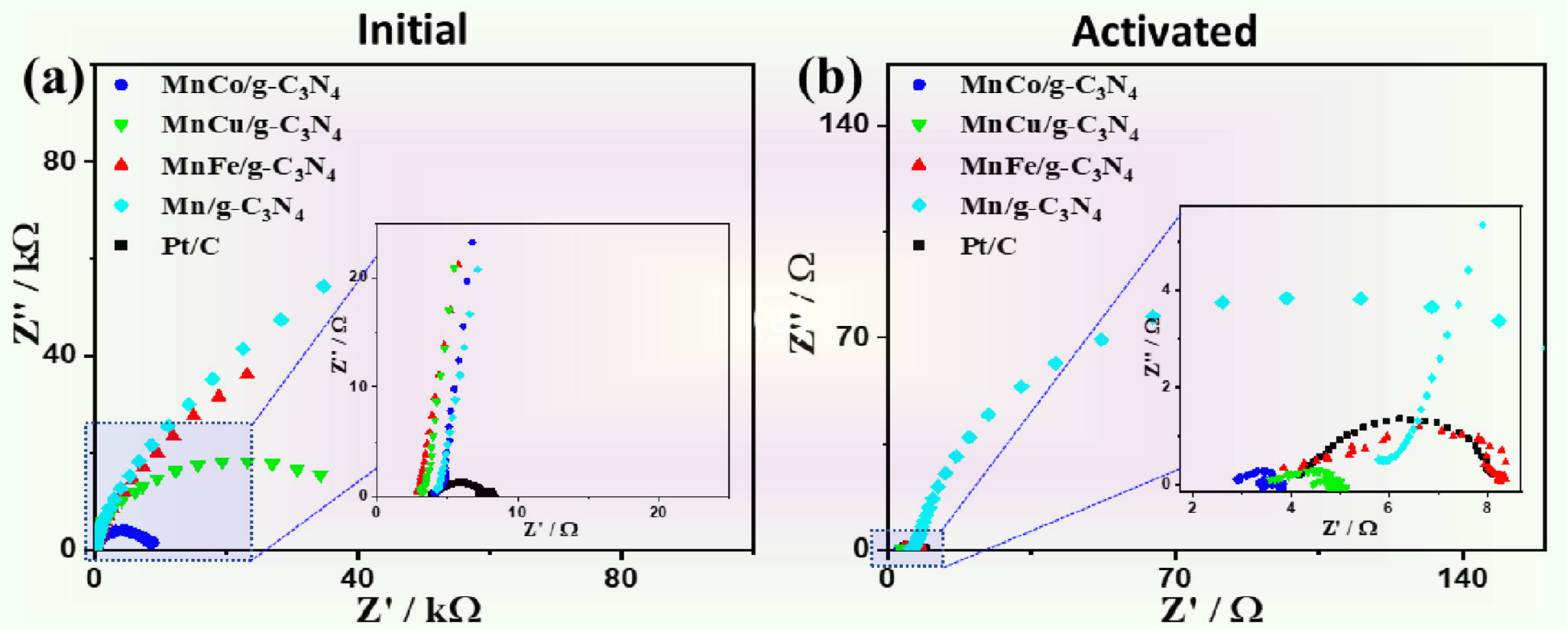
The EIS Nyquist plots of a) non‐activated and b) activated MnM/g‐C3N4 relative to Pt/C measured at 15 mV. The inlets in a) and b) magnify the selected areas marked by the box.

Electrochemical impedance spectroscopy (EIS) measurements before and after the reductive activation display a significant decrease of the charge transfer resistance on the catalyst surface by four orders of magnitude (i.e., from 4.23 kΩ to 0.29 Ω on MnCo/g‐C_3_N_4_) (**Figure** **5**). The fitting of EIS data validates the lower electrolyte resistance (*R*_s_) and charge transfer resistance (*R*_ct_) after activation in general, with the lowest values on MnCo/g‐C_3_N_4_ (Table S6, Supporting Information). Notably, the fitted constant phase elements (CPEs) increased by a factor of more than 20 after activation. This all due to the reduction of the catalyst under negative bias potentials, and a metal‐like electronic conductivity, practically free of impedance, is also provided across the phase boundaries.

After chronoamperometry for 15 days at 7 mV, MnCo/g‐C_3_N_4_ maintained 99.8% of its initial activity. Polarization cycles were run for 10 000 cycles, and the onset potential ƞ_HER_ increased by only (1.5 mV), a value even minor when compared to Pt/C (17 mV) (Figures S9 and S10, Supporting Information). This indicates a close‐to‐perfect stabilization of active sites and transport pathways within the MnCo/g‐C_3_N_4_ electrocatalyst. As one can hardly find metal single atom catalysts with similar stability, we consider this also as proof that the covalent g‐C_3_N_4_ structure is the catalyst as such. The TEM images on the sample measured after 10 000 polarization cycles show an apparently unchanged nanostructure, too, confirming the electrochemical data (Figure S11a, Supporting Information). Meanwhile, EDX analysis displays compositional durability as shown in preserving the atomic content without any loss in C/N/Mn/Co (Figure S11b, Supporting Information).

**Figure 6:**
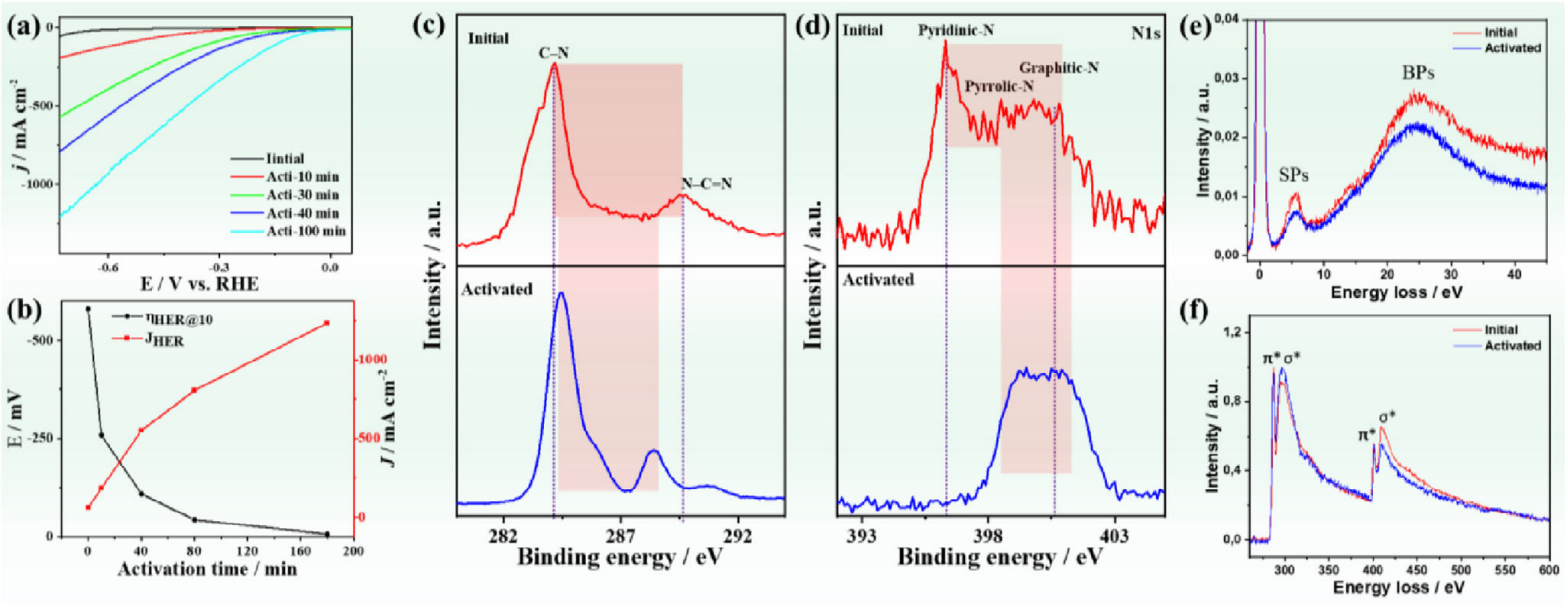
The LSV of MnCo/g‐C_3_N_4_ was measured in 0.5 M H_2_SO_4_ at 2 mV s^−1^ before and after reductive activation for various durations a), along with the relevant changes in *J_HER_* versus *η_HER_*_@10_ b). High‐resolution XPS spectra of c) C 1s and d) N 1s of MnCo/g‐C_3_N_4_ were recorded before and after activation for 3 h. Reflection EELS spectra e) and EELS spectra of carbon and nitrogen f) of MnCo/g‐C_3_N_4_ before and after activation.

To elucidate the reductive activation mechanism, we conducted a series of electrochemical and spectroscopic analyses. The HER performance of M/g‐C_3_N_4_ (where M represents Mn, Fe, Co, and Cu) was evaluated both before and after activation. The results demonstrated a significant enhancement in HER activity for all catalysts after activation, with an increase of at least ten times. The LSV tested under various activation times (i.e., from 10 min to 3 h), showed that the reduction is rather slow and saturates into a maximum hydrogen loading with time, and as a rule of thumb, 3 h activation with −0.6 V bias potential is sufficient to reach the optimum of reactivity (**Figure** **6**a). This is seen in boosting the current density from 60 mA cm^−2^ to 1.3 A cm^−2^ and lowering η_10_ from 579 to 7 mV (Figure 6b).

XPS after activation revealed pronounced narrowing of C 1s and N 1s peaks, an increased C–N/N–C═N ratio, and opposite shifts of C–N (to higher) and N–C═N (to lower) binding energies‐signatures of electron transfer to the carbon sites along the structural pore site while the next neighbor bridge nitrogens compensate the charge within the covalent structure (Figure 6c,d). In the N 1s spectra, narrowing and a reduced pyridinic‐N intensity were observed, indicating electron redistribution within the then hydrogenated g‐C_3_N_4_ to a new conjugation motif. All that involves practically all carbon and nitrogen atoms, that is, the electron transfer is too high to be explained by the ≈2 wt.% single metal atoms.

The reflection low‐energy‐loss EELS spectra recorded before and after activation showed clear peaks corresponding to surface plasmons (SPs) and bulk plasmons (BPs). However, both peaks exhibited a noticeably lower intensity and a slight shift following activation (Figure 6e), attributable to a perturbed band structure due to H*‐addition. Additionally, the bandgap became narrower after activation^[^ ^20^ ^]^ (Figure S13a, Supporting Information). Noticeably, the EELS spectra in the binding region of the C and N K‐edge show a significant change (Figure 6f). After activation, the C K‐edge spectra showed a reduced π*/σ* intensity ratio, indicative of a diminished role of π − conjugation relative to the initial state (Figure S13b, Supporting Information). In contrast, the N K‐edge spectra exhibited an increased π*/σ* ratio, suggesting an increasing π‐character of nitrogen species (Figure S13b, Supporting Information). This distinct behavior can be explained by an alteres conjugation pattern after the massive reduction of the aromatic system, very similar to the low molecular weight model system of flavine. Density functional theory (DFT) calculations were extensively applied to carbon nitride systems, both for metal single atom modifications, but the focus was mainly on the photocatalytic applications^[^ ^7^, ^22^ ^]^ and did not include massive chemical reduction. This is to be analyzed in forthcoming work, also including oxidation reactions.

## 3 Conclusion

To summarize, we presented here the reductive activation of scrolled g‐C_3_N_4_ nanostructures made by single metal atom‐promoted exfoliation. Interestingly, the metal single atoms charged the nanostructure of the carbon nitride to 2D sheets, which then rolled up to scrolls with expanded graphitic layer stacking, which turned the system sensitive toward reductive activation and integration of very high amounts of proton‐electron pairs. The HER performance of MnM/g‐C_3_N_4_ was superior to g‐C_3_N_4_, with lowered overpotential and boosted J_HER_, TOF, and H_2_ production rate. The HER activity of MnCo/g‐C_3_N_4_ after activation changes from weakly active to best‐in‐class, and the optimized reduced structure outperformed even the gold‐standard of a Pt/C catalyst. The ƞ_HER_ and Tafel slope of MnCo/g‐C_3_N_4_ improved, while the *J_HER_* and TOF increased by 25 and 80 times, respectively. This activity could be maintained at 99.8% of the primary hydrogen generation rate after two weeks of operation, and the durability was confirmed to be higher than 10 000 polarization cycles without affecting electrochemical behavior, morphology, and composition. The special structure of the rather open scrolls provides an optimized electron‐proton conduction system to the catalytic sites, where the final liberation of H_2_ takes place in a two‐electron process, as indicated by the Tafel slope. Similarly, exciting, we observe below the onset potential of hydrogen liberation a preloading of the structures with proton‐electron pairs by intercalation up to extremely high levels, which can reach in the present set of experiments 1 electron per 60 mass units of the overall structure. This value is, for instance, higher than the values reached by Lithium intercalation in graphite (1 electron per 79 mass units), and it can be also understood as the realization of an effective “proton battery”. The sum of all these properties allowed us to present MnCo/g‐C_3_N_4_ as an affordable, noble metal‐free electrocatalyst that works at productivities higher than 1.3 A cm^−2^ with lower electric losses and higher durability than the previous reference catalyst, such as commercial Pt/C. By utilizing g‐C_3_N_4_ for electrocatalysis and avoiding noble metals, one might conclude that the sustainable conversion of stranded electricity into energy storage molecules can enter a new phase.

The gap between fundamental discovery and application is however to be addressed by optimizing the catalysts in flow cells and proton exchange membrane electrolyzers and under the parameters related to them (e.g., high flow rates, concentrated electrolytes, and elevated temperatures) in ongoing and future research.

## Conflict of Interest

The authors declare no conflict of interest.

## Author Contributions

K.E. and M.A. contributed equally to this work.

## Supporting information



Supporting Information

## Data Availability

The data that support the findings of this study are available from the corresponding author upon reasonable request.
