# Supplementary material for: Water Electrolysis Beyond Platinum: Carbon Nitride Materials for Electrochemical Hydrogen Evolution
Source: Adv Sci (Weinh). 2025 Sep 23;12(45):e14030. doi: 10.1002/advs.202514030 (PMC12677623; doi:10.1002/advs.202514030)
Supplement: Supplementary file 1 — Supporting Information [file ADVS-12-e14030-s001.docx]

**Water Electrolysis Beyond Platinum: Carbon Nitride Materials for electrochemical Hydrogen Evolution**

*Kamel Eid^1*^ and Markus Antonietti ^1*^*

1. Department of Colloid Chemistry, Max Planck Institute of Colloids and Interfaces, MPI Research Campus Golm, D-14424 Potsdam-Golm, Germany

*Corresponding authors: antonietti@mpikg.mpg.de and Kamel.Eid@mpikg.mpg.de

**Experimental Section**

**Chemicals**

Manganese (II) chloride monohydrate (MnCl_2_·H_2_O), copper (II) chloride ((CuCl_2_.2H_2_O), 99.99 %), cobalt (II) chloride ((CoCl_2_.2H_2_O), 99.99 %), (melamine, 99 %), Iron (II) chloride tetrahydrate (FeCl_2_ · 4H_2_O), **ethanol solution ((CH_3_CH_2_-OH), 99.8 %)**, commercial Pt/C catalyst (10 wt.% Pt), and nitric acid ((HNO_3_), 70 %) were purchased from Sigma-Aldrich Chemie GmbH.

**Synthesis of MnCu/g-C_3_N_4_**

MnCo/g-C_3_N_4_ porous necklace-like nanostructures were synthesized according to our previous reports(*1, 2*), but with slight modifications comprising the direct mixing of melamine (4 g) in an aqueous solution of methanol (200 mL) containing MnCl_2_.2H_2_O (10 mM) and CoCl_2_.H_2_O (10 mM) under magnetic stirring at 25 ^o^C for 20 min. An aqueous solution of 30 mL HNO_3_ (0.2 M) was then added dropwise under magnetic stirring (100 rpm). The precipitate was collected via vacuum filtration using a Whatman Nuclepore Hydrophilic Membrane (0.1 µm Pore Size, 50 mm) and washed with ethanol/H_2_O (3/1 v/v) 3 times to remove impurities. Finally, the obtained powder was dried at 100 °C for 1 h and then annealed at 550 °C (5^o^/min ramping rate) for 2 h under N_2_.

MnCu/gC_3_N_4_ and MnFe/g-C_3_N_4_ necklace-like nanostructures were synthesized using the same method as MnCo/g-C_3_N_4_ but replacing CuCl_2_.2H_2_O with (CuCl_2_·H_2_O) and (FeCl_2_·4H_2_O); respectively. Meanwhile, Mn/gC_3_N_4_ necklace-like was prepared by the same method as MnCo/g-C_3_N_4_ but using only MnCl_2_.2H_2_O (10 mM), and pristine g-C_3_N_4_ was synthesized in the absence of metal precursors.

**Materials Characterization**

The imaging and bulk composition were analyzed on a transmission electron microscope ((TEM), TecnaiG220, FEI, Hillsboro, OR, USA), equipped with high-angle annular dark-field scanning transmission electron microscopy (HAADF-SEM), and energy dispersive spectrometer (EDX). The scanning electron microscope (SEM) was conducted on (SEM, Hitachi S-4800, Hitachi, Tokyo, Japan)). The valence state and surface composition were analyzed with a X-ray photoelectron spectroscopy (XPS) spectra and measured on a Thermo ESCALAB 250 spectrometer. The crystallinity and structure phases were carried out on an X-ray diffractometer (X'Pert-Pro MPD, PANalytical Co., Almelo, Netherlands). The inductively coupled plasma optical emission spectrometry determined the bulk composition and metal loading on the working electrode (ICP-OES, [Agilent 5800](https://www.agilent.com/en/products/icp-oes/icp-oes-instruments/5800-icp-oes-instrument)). The N_2_-adsorption/desorption isotherms were recorded using a Quantachrome Autosorb 3.01 instrument to measure the surface area by Brunauer-Emmett-Teller (BET) and pore size/volume by Barrett, Joyner, and Halend (BJH).

**Electrochemical HER**

The HER performance was conducted using cyclic voltammograms (CVs), linear sweep voltammograms (LSV), impedance spectroscopy (EIS), and chronoamperometry (CA) test on Gamry potentiostat (Reference 3000, Gamry Co., Warminster, PA, USA) using a three-electrode cell of Pt wire (3mm) (counter electrode), Ag/AgCl (in 0.4 M KCl, reference electrode), and carbon foam (1 x 0.5 cm, working electrode). The carbon foam electrodes were initially cleaned by consecutive washing cycles with acetone/water (2/1, v/v ratio), ethanol/water(2/1, v/v ratio), and water for 30 min each at room temperature and then dried in oven under vacuum at 80 ^o^C for 1 h before coating with catalyst ink. The catalyst inks were prepared by dispersion of the catalysts (2 mg) in an aqueous solution of isopropanol/H_2_O/Nafion (5 wt. %) (4/1/0.05 v/v/v). The carbon foam electrodes were coated with 10 µL of the catalyst ink through volumetric casting and then left to dry in an oven at 80 ^o^C for 2h before utilization. The obtained HER currents were normalized to the geometric area of the working electrode after correction against Ohmic potential drop; meanwhile, the potentials were normalized to the reversible hydrogen electrode (RHE) using Eqn. 1

E_RHE_ = E_Ag/AgCl_ + 0.197 V + 0.059 X pH (Eqn. 1)

The turnover frequency (TOF) (s^-1^) was calculated using Eqn. 2

TOF=*J*.A/2Fm (Eqn. 2)

Where *J* is the current density (A.m^2^), A is the area (m^2^), F is the Faraday constant 96,485.33289 (C.mol^-1^), and m is the number of moles of metals.

All the LSV, chronoamperometry, and EIS electrocatalytic tests were performed on the electrodes under stirring at 350 rpm to remove any accumulated bubbles from the surface of the electrodes.

**Supplementary Table 1** Comparison of our activated MnCo/g-C_3_N_4_ catalysts with the previously reported carbon-based materials for HER in the acidic medium.

| **Catalyst** | **Preparation**  **Method** | **E_Onset_ (mV)** | **η_10_ (mV_RHE_)** | **Tafel slope [mV dec^−1^]** | **TOF**  **S^-1^** | **Electrolyte** | **Ref.** |
| --- | --- | --- | --- | --- | --- | --- | --- |
| MnCo/g-C_3_N_4_ | Polymerization/annealing | ~ 0 | 7 | 89.5 | 89.5@-0.6V | 0.5M H_2_SO_4_ | Our Work |
| MnCu/g-C_3_N_4_ |  | ~ 0 | 39.4 | 128 | 76.3@-0.6V |  |  |
| MnFe/g-C_3_N_4_ |  | ~ 0 | 52.7 | 145 | 71.9@-0.6V |  |  |
| Cu/g-C_3_N_4_ | Polymerization/ annealing | -244 | 449 | 219 | 3.5@0.4V | 0.5M H_2_SO_4_ | (*2*) |
| Hollow Zn_0.30_Co_2.70_S_4_ | Solvothermal | -35 | 80 | 47.5 | - | 0.5 H_2_SO_4_ | (*3*) |
| CoP@NPCSs | Pyrolysis | -30 | 112 | 70 | - | 0.5M H_2_SO_4_ | (*4*) |
| Co_2_P@NPC/rGO | Hydrothermal | −83 | 137 | 50.64 | - | 0.5M H_2_SO_4_ | (*5*) |
| Ni@NC@MoS_2_ microspheres | Hydrothermal | -18 | 82 | 50.64 | - | 0.5M H_2_SO_4_ | (*6*) |
| CoPS@NPS-C | Pyrolysis | -50 | 93 | 63 |  | 0.5M H_2_SO_4_ | (*7*) |
| Ni-Co-MoS_2_ @NC | Solvothermal | -125 | 155 | 51 |  | 0.5M H_2_SO_4_ | (*8*) |
| Co@N–C-600 | Precipitation method and | -96 | 339 | 119 | - | 0.5M H_2_SO_4_ | (*9*) |
| CoSe_2_@DC | Pyrolysis | -40 | 150 | 82 | - | 0.5M H_2_SO_4_ | (*10*) |
| Cu-Pd/NPCC/HT/GCE | Hydrothermal | -82 | 340 | - | - | 0.5M H_2_SO_4_ | (*11*) |
| CoNi@NCNTs | Solvothermal | -40 | 130 | 82.1 | - | 0.5M H_2_SO_4_ | (*12*) |
| MnFe_2_O_4_/Graphene | Solvothermal | - | 313 | 106.4 | 1.47 | 0.5M H_2_SO_4_ | (*13*) |
| MnFeCu/g-C_3_N_4_ NFs | Polymerization/ annealing | -225 | 400 | 103 | 4.14 | 0.5M H_2_SO_4_ | (*14*) |
| Hybrid g-C_3_N_4_@NG | Physical mixing/lyophilization/annealing | - | 240 | 51.5 | - | 0.5M H_2_SO_4_ | (*15*) |
| g-C_3_N_4_ nanoribbon-G | Hummers method / Hydrothermal | -80 | 207 | 54 | - | 0.5M H_2_SO_4_ | (*16*) |
| g‐ C_3_N_4_@S‐Se‐pGr | Hummers method/ anneling | -92 | 300 | 86 | - | 0.5M H_2_SO_4_ | (*17*) |
| Cu-doped g-C_3_N_4_ | Mixing / Calcination | -260 | 390 | 76 | - | 0.5M H_2_SO_4_ | (*18*) |
| Carbon-rich BCN (BC_7_N_2_) | Mixing/ drying/ annealing | -56 | 70 | 100 | - | 0.5M H_2_SO_4_ | (*19*) |
| g-C_3_N_4_-0.5%CB 1.0%NiS | Pyrolysis / sonochemical | -698 @NHE | - | - | - | 0.5M H_2_SO_4_ | (*20*) |
| CN/BG hybrid | Hummers method / Annealing | -70 | 260 | 90 | - | 0.5M H_2_SO_4_ | (*21*) |
| g-C_3_N_4_ | Pyrolysis / quenching process liquid N_2_ | -560 | 760@ 0.3mA.cm^-2^ | - | - | 0.5M H_2_SO_4_ | (*22*) |
| g-C_3_N_4_ | Pyrolysis/nitrogen plasma  treatment | -150 | 353 | 83.6 | - | 0.5M H_2_SO_4_ | (*23*) |
| 0.50% C_3_N_4_CNFs | Calcination/electrospinning | -110 | 206 | 106 | - | 0.5M H_2_SO_4_ | (*24*) |
| MGCN | Mixing/annealing | -197 | 272 | 101 | - | 0.5M H_2_SO_4_ | (*25*) |
| g-C_3_N_4_  CN-S(3:2) | Hydrolysis / thermal treatment | -120 | 258 | 109 | - | 0.5M H_2_SO_4_ | (*26*) |
| 3DG/g-C_3_N_4_/Cu_3_P(3DG-Mix) | CVD/ calcination / Mixing/ casting | -5 | 67 | 45 | - | 0.5M H_2_SO_4_ | (*27*) |
| 1%Pt/UH-g-C3N4 | Hydrothermal / calcination | -350 | 450 | 85.3 | - | 0.5M H_2_SO_4_ | (*28*) |
| TE_DCN, TE_UCN, and TE_MCN | calcination / thermal exfoliation | ∼-90  ∼-100  ∼-180 | 220  300  400  @4mA | 78  92.4  104.8 | - | 0.5M H_2_SO_4_ | (*29*) |
| 10%C_60_ /BCN | Mixing / carbonization / sonication | -42 | 222 | 87 | 2.63 | 0.5M H_2_SO_4_ | (*30*) |
| mp-gCN @3DG | calcination / HF etching/casting | -4.2 | 95 | 98 | - | 0.5M H_2_SO_4_ | (*31*) |
| Pt/P-C_3_N_4_ | Annealing / thermal  refluxing | - | 22 | 31.2 | - | 0.5M H_2_SO_4_ | (*32*) |
| Ru-g-CN and Ir-g-CN | thermal  decomposition/ calcination / solvothermal | - | 54.5  41.9 | 54  63.5 | 12.9@0.1V | 0.5M H_2_SO_4_ | (*33*) |
| ternary Ti_3_C_2_T_x_ /g-C_3_N_4_ /RGO | Hummers’method / etching/liquid-phase exfoliation / co-assembly | -38 | 148 | 76 | - | 0.5M H_2_SO_4_ | (*34*) |
| g-C_3_N_4_/rGO | thermal reduction / calcination /  electrostatic self-assembly method | -150 | 183 | 164 | 0.026@0.3V | 1M KOH | (*35*) |
| Pt_150_@g-C_3_N_4_ | Annealing/dissolving and drying | -20 | 44 | 67 | 37.2@0.05V | 0.5M H_2_SO_4_ | (*36*) |
| GCN/ACN/GO | Hummers’ method/calcination/impregnation/calcination | -90 | 1400 | - | - | 0.5 M Na_2_SO_4_ | (*37*) |
| PCN@N-graphene and  PCN@N-graphene 750 | Stöber’s  Method / Hummers’  Method/annealing/reduction | -11  -8 | 170  80 | 88.6  49.1 | - | 0.1M KOH | (*38*) |
| MPSA/GO-1000  and  MPSA/GO-500 | Hummers method/self-assembly/annealing | -65  -570 | 163  850 | 89  250 | - | 0.1M KOH | (*39*) |
| SHG | Lyophilization / pyrolysis | -230 | 310 | 112 | - | 0.1M KOH | (*40*) |
| g-CN@G MMs | Hydrothermal / freeze drying/calcination | -78 | 219 | 53 | - | 0.5M H_2_SO_4_ | (*41*) |
| SCN-MPC | pyrolysis / HF etching/polycondensation/annealing | -60 | 145 | 51 | - | 0.5M H_2_SO_4_ | (*42*) |
| mesoporous g-C_3_N_4_ (C-MGCN-0.3) | Hydrothermal / carbonization/template | -160 | 314.8 | 30 | - | 0.5M H_2_SO_4_ | (*43*) |
| BDC-NH_2_@Co−C_3_N_4_ | Impregnation / calcention / solvothermal | -100 | 198 | 99 | - | 0.5M H_2_SO_4_ | (*44*) |

**Formation mechanism**

The protonation and subsequent polymerization process over the course of the reaction time could be monitored by the naked eye, seen as changing the color of the melamine solution from a milky dispersion to precipitates with different colors (i.e., pink, seaweed, and red) after the addition of HNO_3_ (**Figure** **S1)**. The changing color of the precipitates is attributed to the polymerization of melamine and integration of the two metal ions. Finally, the obtained powder turned to yellow color after complete annealing.


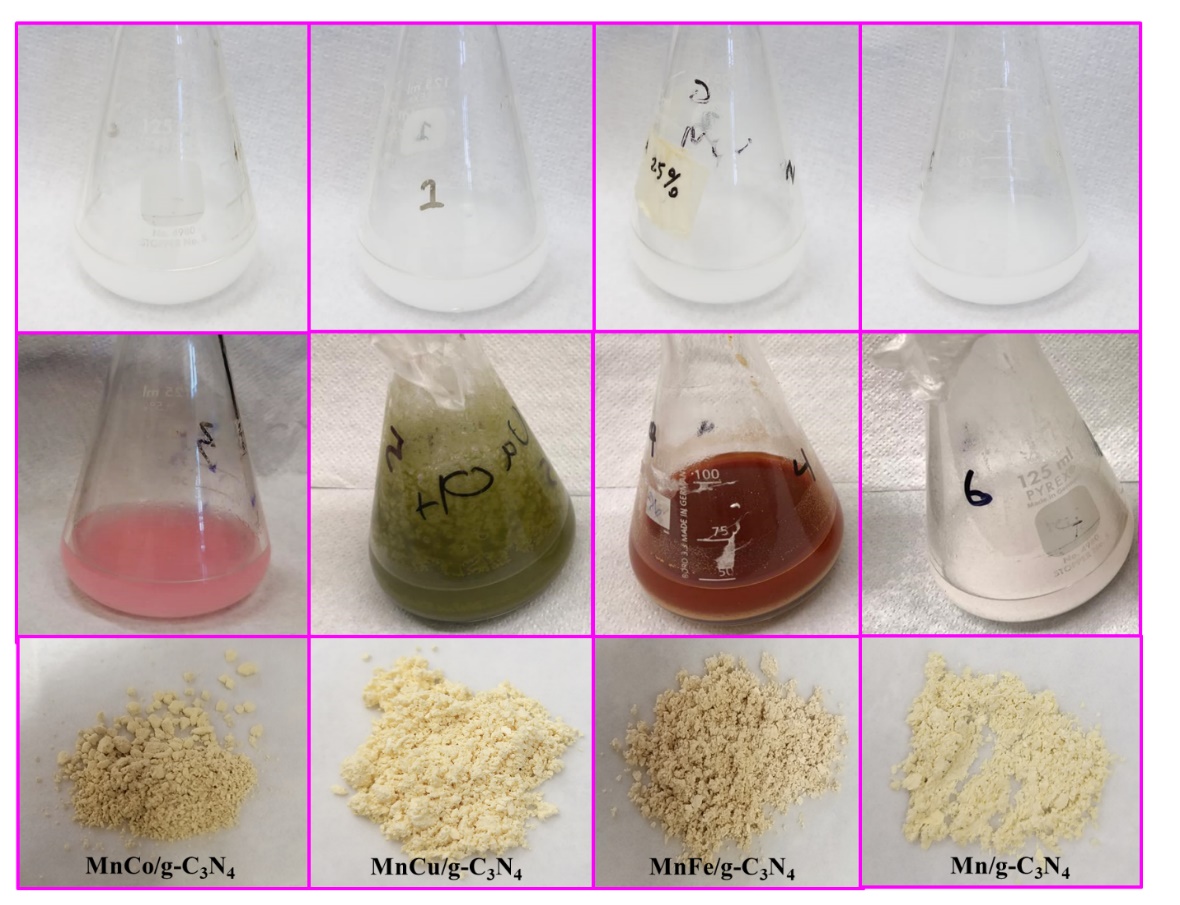


**Supplementary Figure 1** The precipitates formed after the addition of nitric acid and the final annealed powder.


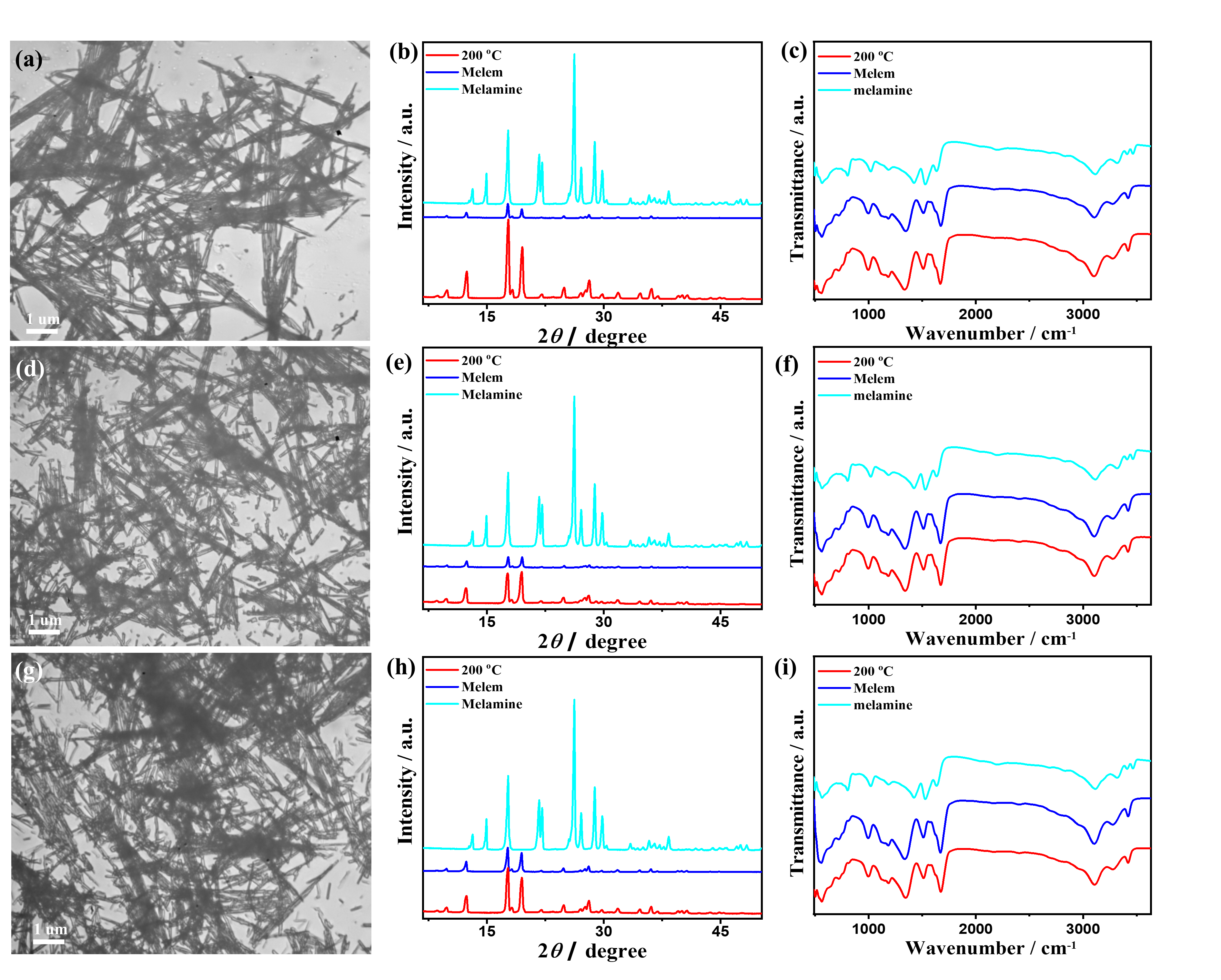


**Supplementary Figure 2** SEM, XRD, and FTIR for (a) MnCo/g-C_3_N_4_, (b) MnCu/g-C_3_N_4,_ and (c) MnFe/g-C_3_N_4_.

The obtained precipitates that obtained after the addition of HNO_3_ to melamine in an aqueous solution of metal slats were collected and analyzed by the SEM, XRD, and FTIR to confirm the formation mechanisms. The sequential formation of melamine to melem is monitored by the SEM images, XRD, and FTIR analysis, which implies the direct transformation of melamine irregular crystal melamine powders to melem in one-dimensional fiber-like structure after the addition of HNO_3_ **(Figure 2)** at room temperature, then melone after heating to ≥300 ^o^C, and finally to g-C_3_N_4_ at 550 ^o^C. The SEM image confirmed the formation of porous one-dimensional ultra-long fiber-like structure, which implies the rolling-up mechanism occurs under stirring at room temperature (Figure S2a, d, g).  The XRD and FTIR peaks of the obtained intermediates showed the conversion of melamine to melem at room temperature, but after annealing the precipitates at 200 ^o^C, the XRD peaks became more intense **(Figure 3).** This implies the formation of melem at a low temperature via the protonation of NH_2_ in melamine by H of HNO_3_ and subsequent eliminating NH_3_. The absence of any change in the XRD and FTIR peaks after annealing at 200 ^o^C, implying that melone only forms at high temperatures higher than 200 ^o^C, as reported before (*45-48*).


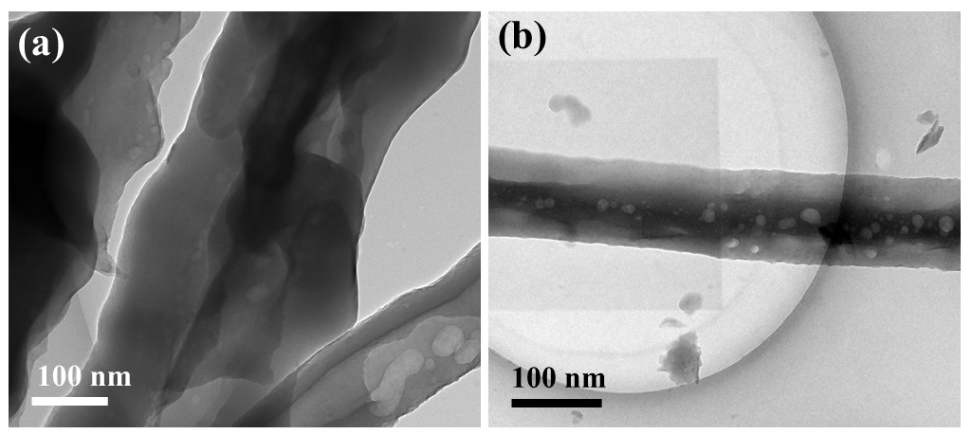


**Supplementary Figure 3** TEM for the as-formed metal-free g-C_3_N_4_ and Mn/g-C_3_N_4_.

Metal-free g-C_3_N_4_ and Mn/g-C_3_N_4_ show porous fiber-like nanostructures **(Figure 3)** with an average length/width of 6µm/106±2 nm and 5.5µm/100±3 nm, but with more porosity than the MnM/g-C_3_N_4_ nanostructures.

**Supplementary Table 2** The composition analysis of the as-synthesized materials

| **catalyst** | **EDX (wt.%)** | | | | **ICP (wt.%)** | | | | **XPS (wt.%)** | | | |
| --- | --- | --- | --- | --- | --- | --- | --- | --- | --- | --- | --- | --- |
|  | Mn | M | C | N | Mn | M | C | N | Mn | M | C | N |
| MnCo-g-C_3_N_4_ | 0.99 | 0.89 | 41.21 | 5 6.91 | 1. 05 | 0.87 | 4 1.07 | 5 7.01 | 1.1 | 0. 92 | 4 1.08 | 5 6.9 |
| MnCu-g-C_3_N_4_ | 0.9 | 0.78 | 41.12 | 5 7.2 | 0. 96 | 0.83 | 4 1 | 5 7.21 | 0.98 | 0. 91 | 4 0.9 | 5 7.21 |
| MnFe-g-C_3_N_4_ | 0.59 | 0.67 | 41.32 | 5 7.42 | 0. 65 | 0.75 | 4 1.26 | 5 7.34 | 0.63 | 0. 72 | 4 1.27 | 5 7.38 |
| Mn-g-C_3_N_4_ | 1.7 | - | 41.4 | 5 6.9 | 1. 58 | - | 4 1.58 | 5 6.84 | 1.52 | - | 4 1.58 | 5 6.9 |


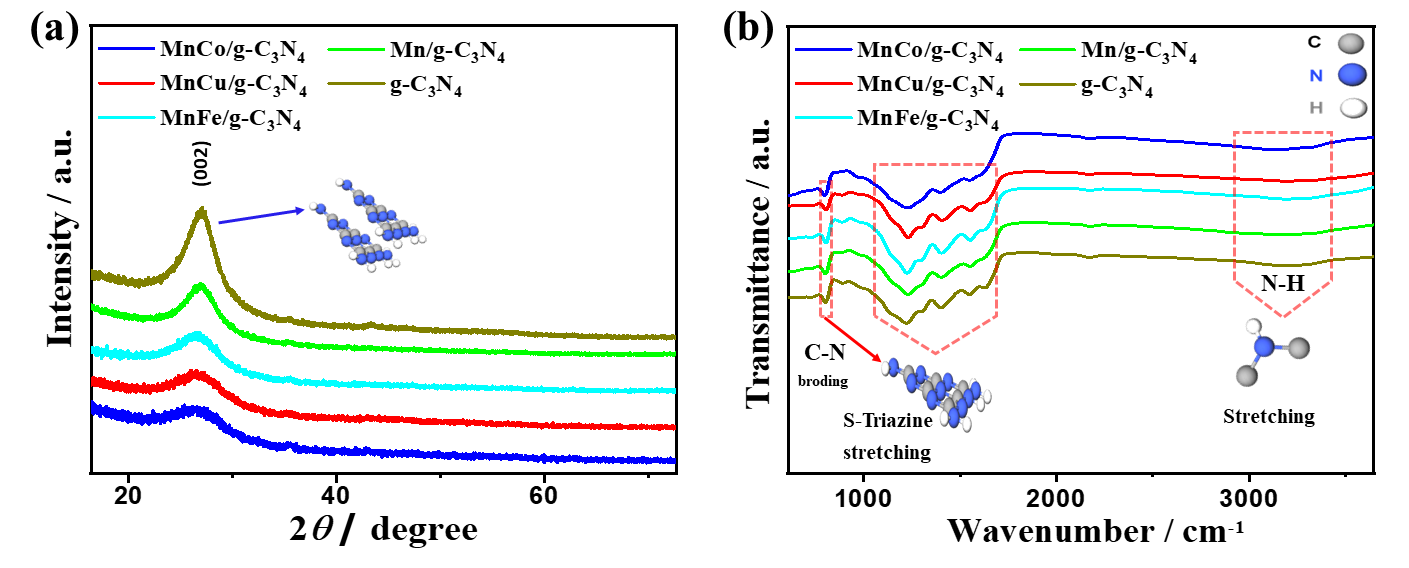
 **Supplementary Figure 4 (a)** XRD analysis and (b) FTIR spectra of MnM/g-C_3_N_4_, Mn/g-C_3_N_4_, and g-C_3_N_4_

**XRD and FTIR Characterizations**

The XRD spectra of MnCo/g-C_3_N_4_, MnCu/g-C_3_N_4_, MnFe/g-C_3_N_4_, Mn/g-C_3_N_4_, and g-C_3_N_4_ depict only one broad diffraction peak at nearly 2*θ* angle of 26.6° that is ascribed to the (002) reflection of interlayer stacking in the g-C_3_N_4_ structures(*49*) (Figure 4a). The peak is expected to be broad because scrolls are missing long-range translational order. The absence of XRD diffraction peaks for pure metal dopants or their oxides (i.e., Cu, Mn, and Fe) is ascribed to their single atom dispersion at low loading amounts of MnM into the skeleton structure of g-C_3_N_4_, in line with similar reports (*1, 2, 18, 50, 51*). The XRD diffraction peak of (002) facet of MnM/g-C_3_N_4_ and Mn/g-C_3_N_4_, is broadened and has a slightly low 2*θ* angle shift relative to bare g-C_3_N_4_, which is possible due to the insertion of metal atoms between the layers in the inner vacancies of g-C_3_N_4_, leading to stress/strain within the crystal lattice causing lattice expansion. This is due to the difference in the atomic radii, electronegativity, and electronic structure between metal atoms and C/N in g-C_3_N_4_, which induce changes in full peak width at half maximum (FWHM), crystallite size, microstrain, and d-spacing (Table 3). This is seen in the increasing FWHM of MnCo/g-C_3_N_4_ (5.188 ^o^), MnCu/g-C_3_N_4_ (5.167 ^o^), MnFe/g-C_3_N_4_ (5.048 ^o^), than Mn/g-C_3_N_4_ (3.528 ^o^), and g-C_3_N_4_ (3.266^o^), due to lattice distortion, defects, and internal stresses **induced by chemical interaction between metal dopants and** g-C_3_N_4_**, which** can lead to different interactions with the reactants and subsequent dissimilar ability to activate and dissociate water in electrolyte during the HER on MnM/g-C_3_N_4_. **This is reflected in the higher** microstrain of MnCo/g-C_3_N_4_ than those of MnCu/g-C_3_N_4_, MnFe/g-C_3_N_4_, Mn/g-C_3_N_4_, and g-C_3_N_4_. Additionally, the d-spacing was in the order of MnCo/g-C_3_N_4_> MnCu/g-C_3_N_4_> MnFe/g-C_3_N_4_> Mn/g-C_3_N_4_> g-C_3_N_4_.

**Supplementary Table 3** The obtained XRD parameters of thus synthesized MnM/g-C_3_N_4_ and Mn/g-C_3_N_4_ materials compared with g-C_3_N_4_. The d-spacing (d, Å), Crystallite size (D, Å) using the Scherrer equation, Strain (ε, %) using Williamson–Hall approach: -

$d=\frac{\lambda}{2\sin\theta}$ , $D=\frac{K\lambda}{\beta\cos\theta}$, $\varepsilon= \frac{\beta}{4\tan\theta}$

Where, X-ray wavelength (λ): 1.5406 Å (Cu Kα), Shape factor (K): 0.9 (common value), β is in **radians.**

|  | 2θ (°) | FWHM (°) | d-spacing (Å) | Crystallite Size (Å) | Strain (%) |
| --- | --- | --- | --- | --- | --- |
| MnCo/g-C_3_N_4_ | 26.53 | 5.188 | 3.357 | 15.73 | 9.60 |
| MnCu/g-C_3_N_4_ | 26.54 | 5.167 | 3.356 | 15.80 | 9.56 |
| MnFe/g-C_3_N_4_ | 26.56 | 5.048 | 3.353 | 16.17 | 9.33 |
| Mn/g-C_3_N_4_ | 26.93 | 3.528 | 3.308 | 23.15 | 6.43 |
| g-C_3_N_4_ | 27.00 | 3.266 | 3.300 | 25.02 | 5.94 |

The FTIR analysis MnM/g-C_3_N_4_, Mn/g-C_3_N_4_, and g-C_3_N_4_ reveal the absorption bands attributed to the breathing mode tris-s-triazine rings at 809 and 889 cm^-1^ in addition to a series of several peaks between 1200-1650 cm^-1^ assigned to the stretching mode of C-N heterocycles. There is also a broad peak between 3000 and 3300 cm^-1^ of the stretching vibration mode of N-H, as usually observed in incompletely condensed or protonated g-C_3_N_4_ (Figure 4b)(*1, 2, 51*). The noticed broadening in the N-H peak in the MnM/g-C_3_N_4_ and Mn/g-C_3_N_4_ can be plausibly attributed to the insertion of metal atoms close to imide positions, which led to a kind of interaction and competition of the metal centers with N-H group (i.e., MnM-N) and a consequent change in the electron density of the N atoms (*1, 2, 51*).


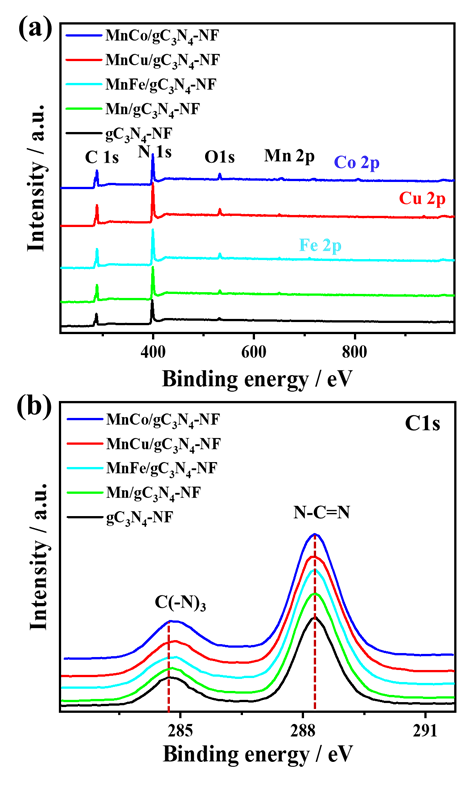


**Supplementary Figure 5** (a) XPS survey and (b) High-resolution XPS of C 1s of MnM/g-C_3_N_4_ relatve to Mn/g-C_3_N_4_ and metal-free g-C_3_N_4_.


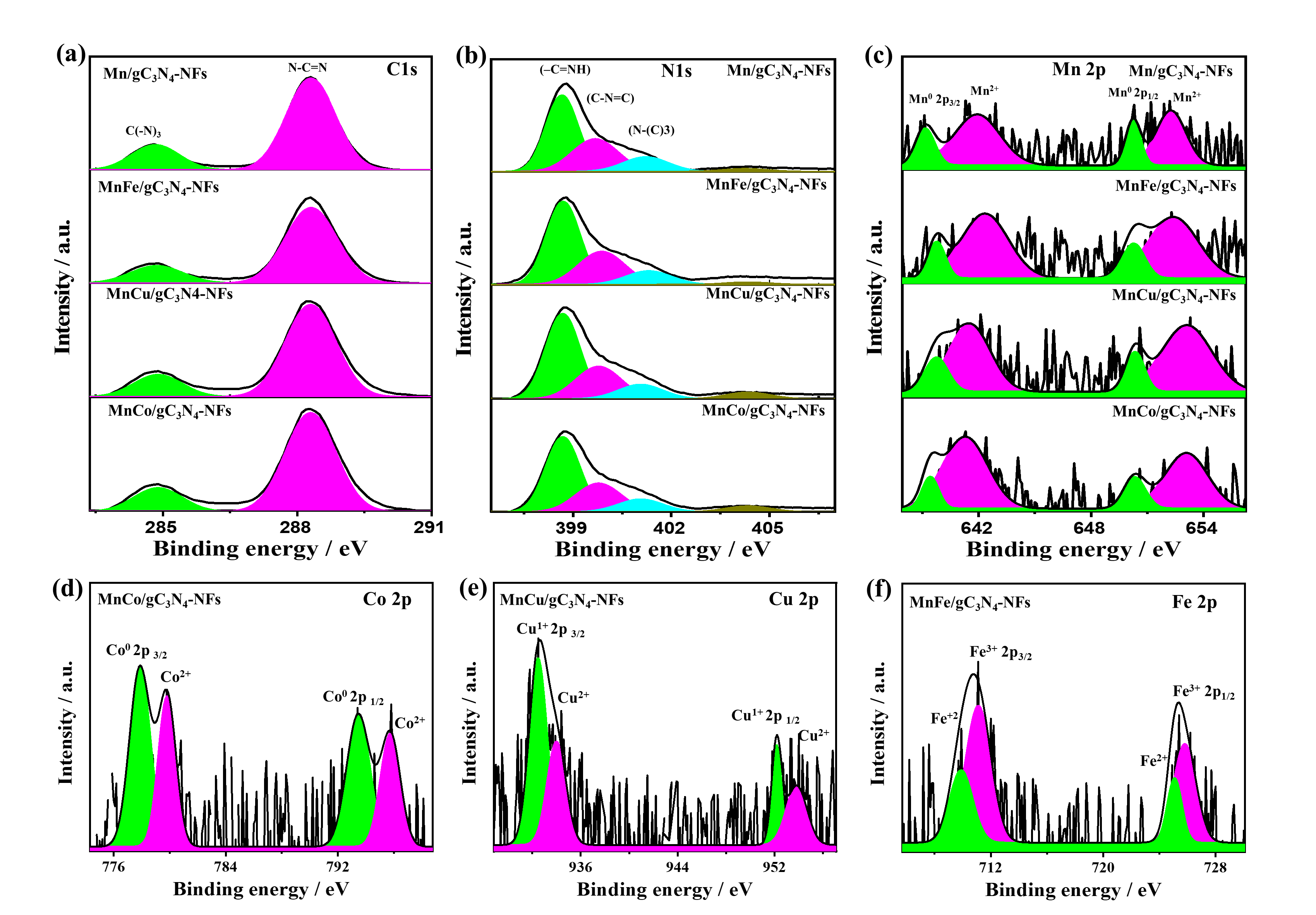


**Supplementary Figure 6.** High-resolution spectra of (a) C 1s, (b) N 1s, (c) Mn 2p in MnM/g-C_3_N_4_, Mn/g-C_3_N_4_, and g-C_3_N_4_. High-resolution spectra of Co 2p of MnCo/g-C_3_N_4_ (d), Cu 2p of MnCu/g-C_3_N_4_ (e), Fe 2p of MnFe/g-C_3_N_4_ (f).

**XPS Analysis**

The XPS survey spectra show the core-level of valence states assigned to C 1s, N 1s, O1s and Mn 2p in the obtained materials besides Co 2p in MnCo/g-C_3_N_4_, Cu 2p in MnCu/g-C_3_N_4_, Fe 2p in MnFe/g-C_3_N_4_, whereas pristine g-C_3_N_4_ reveals only C 1s, O1s, and N1s **(Figure 5a)**. **Table 4** shows the binding energies and valance state of the as-obtained materials. Interestingly, the surface atomic content of Mn/Co, Mn/Cu, Mn/Fe, and Mn are found to be 1.1/0.92 % in MnCo/g-C_3_N_4_, 0.98/0.91 % in MnCu/g-C_3_N_4_, 0.63/0.72 % in MnFe/g-C_3_N_4_, and 1.52 in Mn/g-C_3_N_4_, implies the homogenous distribution of binary metal atoms in the bulk and surface. Notably, the C 1s peaks of (C(-N)_3_ planar trigonal carbon geometry) of MnM/g-C_3_N_4_ are slightly shifted towards higher binding energies than that of Mn/g-C_3_N_4_ and metal-free g-C_3_N_4_, which plausibly originated from the redistribution of electrons and increasing the electron density on N-atoms of C−N=C in g-C_3_N_4_ via the coordinative electron transfer to the metal atoms **(Figure 5b)**. This is also evident in the slight blue shift in the binding energies of N 1s peaks of MnM/g-C_3_N_4_ compared to Mn/g-C_3_N_4_ and metal-free g-C_3_N_4_, which indeed imply the visible influence of MnM doping on the electronic band structure of g-C_3_N_4_ (*52*). Modulating the electron density of C−N=C, possibly leading to high intermolecular charge transfer between MnM and g-C_3_N_4_, which is beneficial for promoting the electrocatalytic merits during the HER. The fitting of the C 1s spectra in MnM/g-C_3_N_4_ shows an intense peak assigned to (N-C=N) at 288.25 eV bond as the prominent peak and a weak peak of C(-N)_3_ planar trigonal carbon geometry at 284.8 eV **(Figure 6a)**, whereas, fitting of N 1s spectra of MnM/g-C_3_N_4_ reveals a dominant peak attributed to N-bonded to C sp^2^ in heptazine rings (-C=NH) at 398.68 eV beside a small shoulder for N-bonded to two carbon atoms (C-N=C) at 399.76 eV in imidic bonds. There is also a signal of N trigonally bonded to sp^2^ carbon atoms (N-(C)_3_) at 401.06 eV of graphitic-like nitrogen **(Figure 6b)**(*53*). The deconvolution of Mn 2p spectra in MnM/g-C_3_N_4_ displays Mn^2+^ (2p_3/2_ and 2p_1/2_ ) and Mn^0^, that considered as a strong argument for the presence of Mn as single atom in g-C_3_N_4_ **(Figure 6c)**(*54*). Notably, the Mn 2p peaks in MnM/g-C_3_N_4_ are slightly shifted towards higher binding energies than that of g-C_3_N_4_, which evidences the partial charge transfer to the carbon nitride support. The fitting of Co 2p spectra **(Figure 6d)** shows Co^2+^ (2p_3/2_ and 2p_1/2_) besides Co^0^; meanwhile, Cu 2p spectra **(Figure 6e)** reveal Cu^+1^ (2p_3/2_ and 2p_1/2_) and Cu^2+^ (*51*). Fe^3+^ (2p_3/2_ and 2p_1/2_) is also the primary phase, and Fe^2+^ is only minor in Fe 2p **(Figure 6f)**. The existence of mixed metal oxidation states is plausibly attributed to the oxidation effect of nitric acid or the high oxophilicity of MnM metals, when exposed to air. The presence of binary metal dopants with various valence states is favorable for accelerating the HER kinetics. Meanwhile, the in-situ released gases (i.e., H_2_ and NH_3_) during the protonation and polycondensation processes of melamine may promote the reduction of MnM ions and ease their trapping in C_3_N_4_ skeleton as single atom(*51*). The obtained elemental composition determined by the XPS, EDX, and ICP is shown in **(Table 2)**.

**Supplementary Table 4** The binding energies and valance state of metals.

| **Elements** | **Mn 2p** | | **Co 2p** | | **Cu 2p** | | **Fe2P** | |
| --- | --- | --- | --- | --- | --- | --- | --- | --- |
| Catalyst | 2p 3/2  (Mn^+2^, Mn^+3^, Mn^+4^) | 2p 1/2 | 2p 3/2  (Co^+3^  , Co^+2^) | 2p ½  (Co^+3^  , Co^+2^) | 2p 3/2  (Cu^+1^  , Cu^+2^) | 2p ½  (Cu^+1^  , Cu^+2^) | 2p 3/2  (Fe^+1^  , Fe^+2^) | 2p ½  (Fe^+1^  , Fe^+2^) |
| **MnCo/g-C_3_N_4_** | 640.25,  642.09,  643.82 | 651.56,  653.14,  654.2 | 780.09,  781.9 | 794.9,  795.78 | - | - | - | - |
| **MnCu/g-C_3_N_4_** | 640.27,  641.67,  643.44 | 651.6,  653.07,  654.6 | - | - | 932.4, 934.2 | 952.63,  955.52 | - | - |
| **MnFe/g-C_3_N_4_** | 640.31,  641.81,  643.21 | 651.7,  652.87,  654.3 | - | - | - | - | 709.95,  711.5 | 721.87,  726.05 |
| **Mn/g-C_3_N_4_** | 640.45,  641.85,  643.21 | 651.84,  653.2,  654.6 | - | - | - | - | - | - |


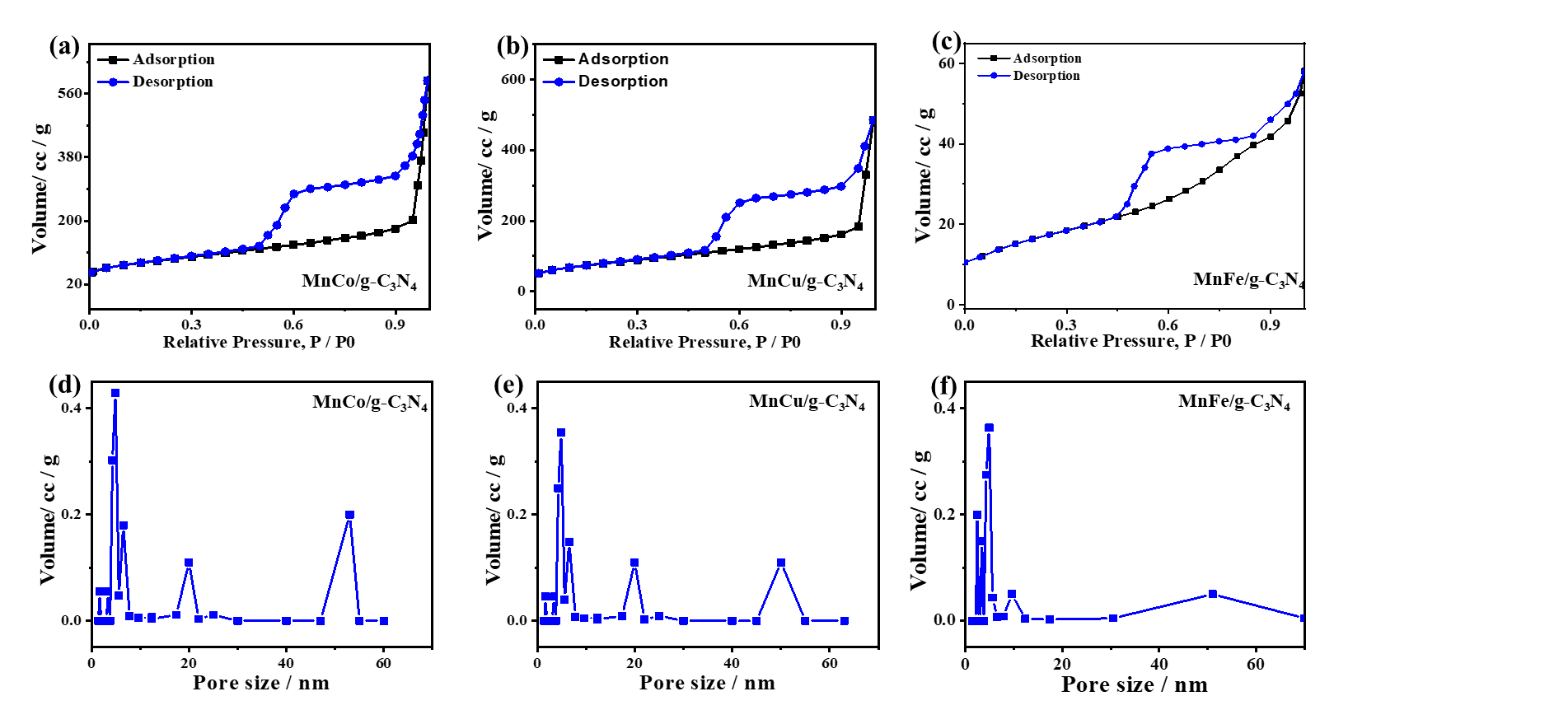


**Supplementary Figure 7.** (a-c) N_2-_adsorption/desorption isotherm, and (d-f) Pore size distribution of MnCo/g-C_3_N_4_ and MnCu/g-C_3_N_4_, MnFe/g-C_3_N_4_ respectively.

**Surface Area and Porosity**

This N_2-ads/des_ isotherm of MnCo/g-C_3_N_4_ and MnCu/g-C_3_N_4_ displays a hysteresis loop close to the type IV curve comprising two-step capillary condensation at P/P_0_≥ 0.5 and P/P_0_ ≤0.99 along with a sudden drop in the desorption curve at 0.6 **(Figure 7a-b)**. This is characteristic of multimodal pore-size distributions (i.e., mesopores and micropores) . The same features were observed in MnFe/g-C_3_N_4_ but with a two-step capillary condensation at P/P_0_≥ 0.45 and P/P_0_ ≤0.99, along with a sudden drop in the desorption curve at 0.55 (Figure 7c). The BET surface areas of MnCo/g-C_3_N_4_, MnCu/g-C_3_N_4_, and MnFe/g-C_3_N_4_ were about 308, 280, and 140 m^2^/g, respectively. The calculated pore diameter of MnCo/g-C_3_N_4_, MnCu/g-C_3_N_4_, and MnFe/g-C_3_N_4_ using Barrett, Joyner, and Halend method ranges from 4-30 nm, and pore volumes of 0.44, 0.36, and 0.25 cm^3^/g, respectively **(Figure 7d-f)**. The pore volume of MnCo/g-C_3_N_4_ (0.44 cm^3^/g) was higher than those of MnCu/g-C_3_N_4_ (0.37 cm^3^/g_ and MnFe/g-C_3_N_4_ (0.3 cm^3^/g ). This goes well with the TEM pictures and the presence of scrolled nanostructures. The porosity is favored for promoting the adsorption of reactants and easing the desorption of products, along with promoting the electrolyte-electrode diffusion and interaction.


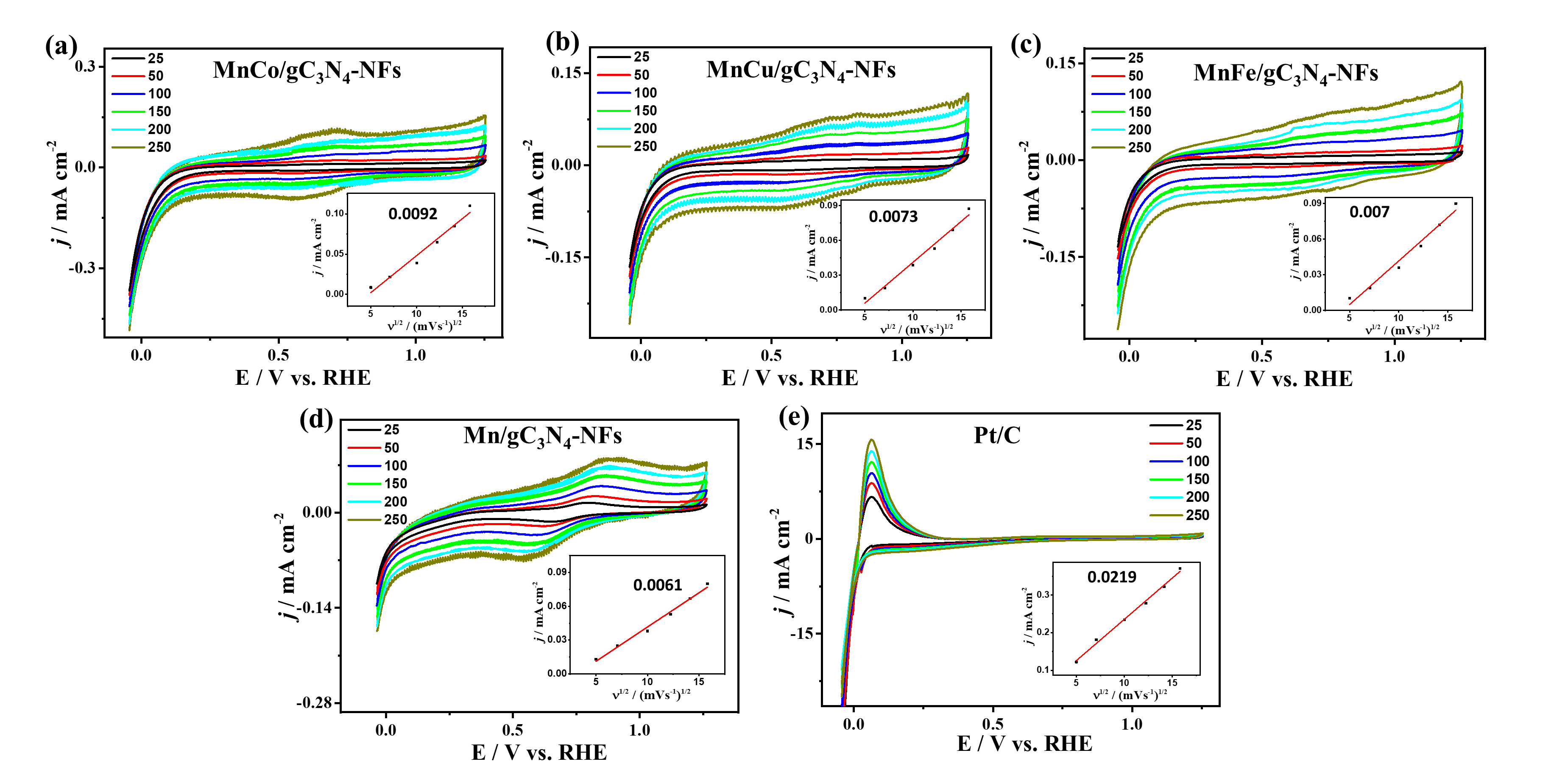


**Supplementary Figure 8.** CV curves at different scan rates and their related plots of *j* vs. v^1/2^ in 0.5 M H_2_SO_4_ for catalysts.

**Supplementary Table 5** The HER activities of MnCo/g-C_3_N_4_, MnCu/g-C_3_N_4_, MnFe/g-C_3_N_4_, Mn/g-C_3_N_4_, and Pt/C catalysts

|  | Catalyst | MnCo/g-C_3_N_4_ | MnCu/g-C_3_N_4_ | MnFe/g-C_3_N_4_ | Mn/g-C_3_N_4_ | Pt/C (10%wt) |
| --- | --- | --- | --- | --- | --- | --- |
| η_10_ (mV) @ 2 mVs^-1^ | Initial | 577 | 614 | 627 | 641 | 7 |
|  | Activated | 7 | 39.4 | 52.7 | 300 | 9 |
| Onset potential (mV) | Initial | -254 | -285 | -301 | -454 | 0 |
|  | Activated | 0 | 0 | 0 | -155 | 0 |
| Tafel (mVdec^-1^) | Initial | 262 | 302 | 320 | 329 | 51.8 |
|  | Activated | 86 | 131 | 133.5 | 136 | 53.6 |
| TOF  (H_2_ S^-1^) @-0.6 V | Initial | 1.1 | 0.88 | 0.82 | 0.79 | 42.3 |
|  | Activated | 89.5 | 76.3 | 71.9 | 25.3 | 42 |
| TOF(H_2_ S^-1^) @-0.1 V | Activated | 8.43 | 3.69 | 2.56 | 0.22 | 5.99 |
| ECSA (cm^2^) | Initial | 29.7 | 23.6 | 22.5 | 19.6 | 69 cm^2^ / 160 m^2^/g |
| HER Rate  @-0.1 V | mol.g^-1^_metal._h^-1^ | 4323 | 1998 | 1354.9 | 118 | 921 |

**Supplementary Table 6** EIS fitting data of MnCo/g-C_3_N_4_, MnCu/g-C_3_N_4_, MnFe/g-C_3_N_4_, Mn/g-C_3_N_4_ and Pt/C before and after activation.

| **Catalyst** |  | ***R*_s_ (Ω)** | ***R*_ct_ (Ω)** | **CPE (μF.s^(1-^*^a^*^)^)** | ***a*** |
| --- | --- | --- | --- | --- | --- |
| MnCo/g-C_3_N_4_ | Initial | 3.1 ± 0.034 | 8690 ± 79 | 20.34 ± 0.29 | 0.945 ± 0.002 |
|  | Activated | 0.86 ± 0.05 | 2.88 ± 0.05 | 480.9 ± 12 | 0.832 ± 0.068 |
| MnCu/g-C_3_N_4_ | Initial | 3.22 ± 0.25 | 40350 ± 540 | 15.79 ± 0.14 | 0.94 ± 0.001 |
|  | Activated | 1.18 ± 0.06 | 3.69 ± 0. 61 | 395.7 ± 21.3 | 0.739 ± 0.06 |
| MnFe/g-C_3_N_4_ | Initial | 4.35 ± 0.023 | 85000 ± 900 | 26 ± 0.19 | 0.901 ± 0.001 |
|  | Activated | 1.4 ± 0.23 | 10.1 ± 0.02 | 355 ± 25 | 0.72 ± 0.023 |
| Mn/g-C_3_N_4_ | Initial | 5 ± 0.053 | 91033± 730 | 10 ± 0.11 | 0.829 ± 0.003 |
|  | Activated | 20 ± 0.12 | 110 ± 2.7 | 51 ± 0.938 | 0.59 ± 0.007 |
| Pt/C (10%wt.) | Initial | 4.4 ± 0.06 | 4.5 ± 0.08 | 335 ± 0.075 | 0.775 ±0.005 |
|  | Activated | 4.1 ± 0.05 | 4.2 ± 0.07 | 312 ± 0.075 | 0.721 ±0.005 |

**
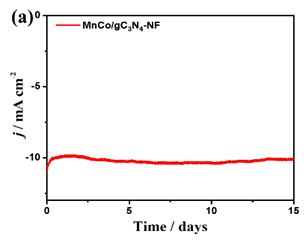
**

**Supplementary Figure 9.** (a) Chronoamperometry stability test of MnCo/g-C_3_N_4_ measured in 0.5 M H_2_SO_4_ at overpotential (7 mV).


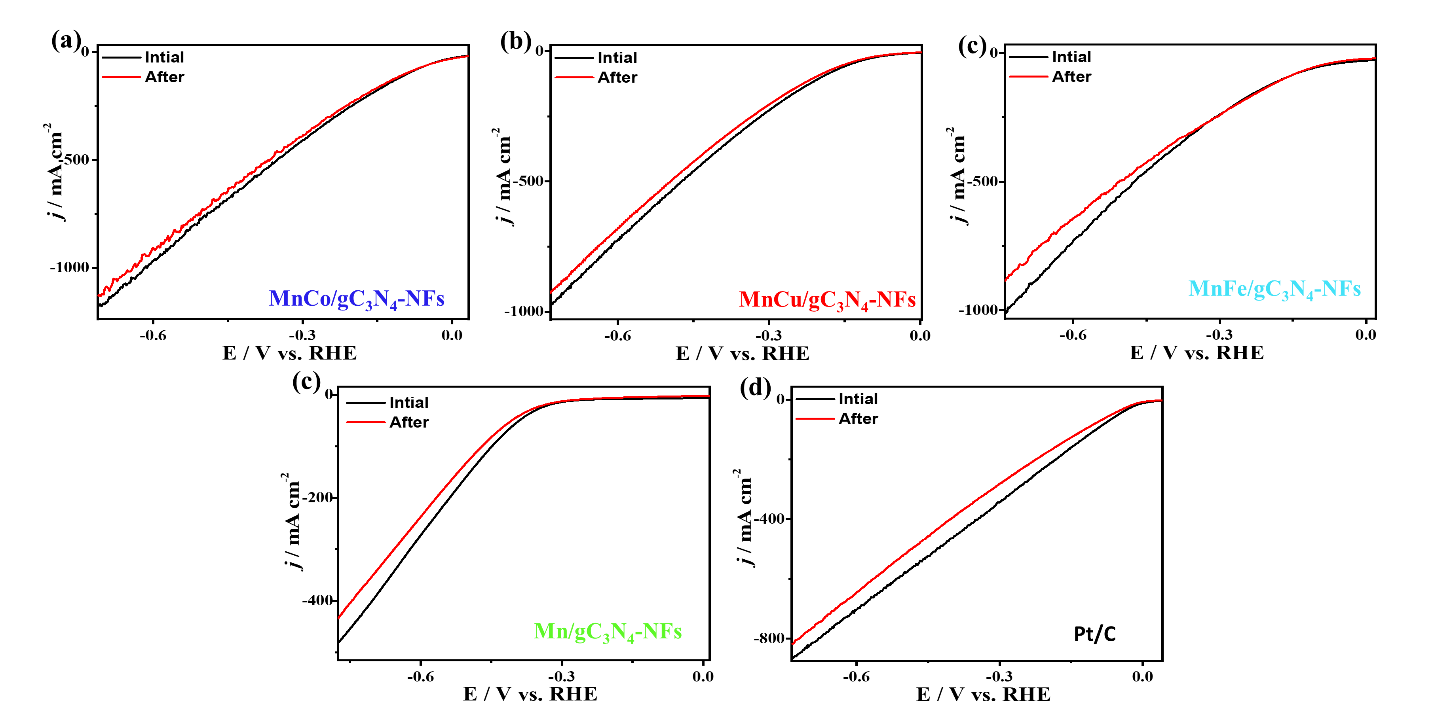


**Supplementary Figure 10.** LSV before and after 10000 cycles measured on (a) MnCo/g-C_3_N_4_, (b) MnCu/g-C_3_N_4_, (c) Mn Fe/g-C_3_N_4_, (d) Mn/g-C_3_N_4_ and (e) Pt/C in an aqueous solution of 0.5M H_2_SO_4_ electrolyte


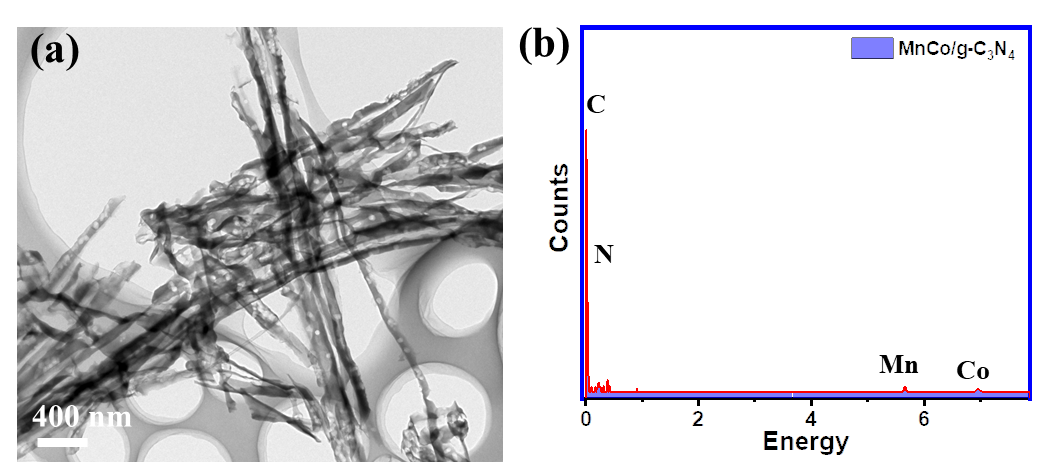


**Supplementary Figure 11** TEM and EDX for MnCo/g-C_3_N_4_ after polarization cycles.


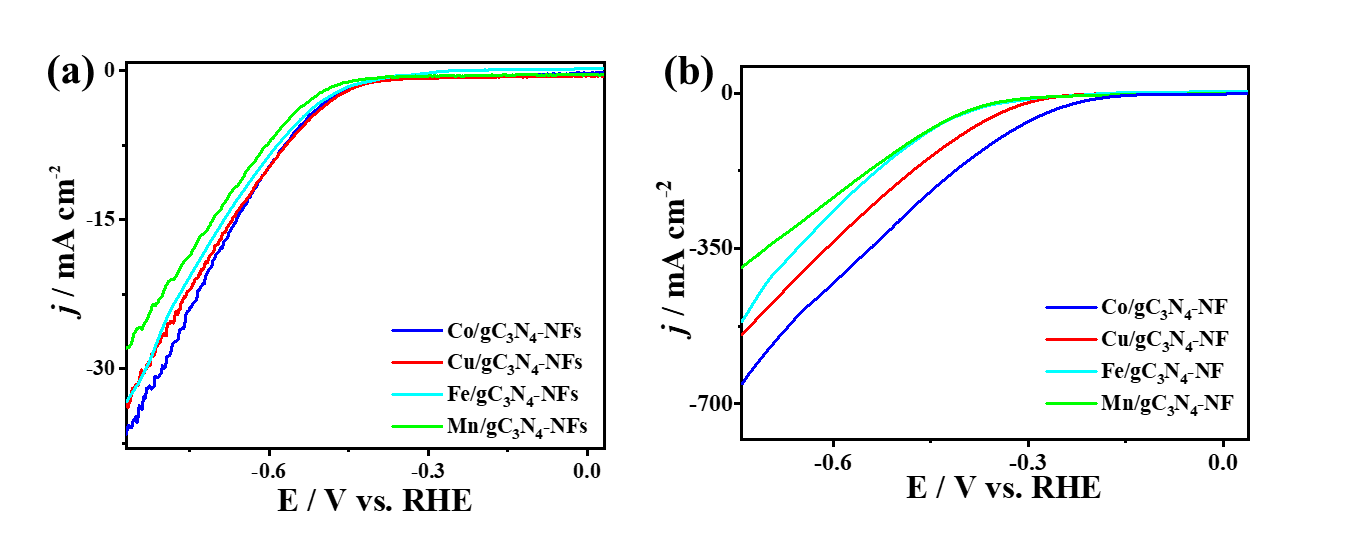


**Supplementary Figure 12** LSV of M/g-C_3_N_4_ (M=Mn, Cu, Co, and Fe) measured in an aqueous solution of 0.5 M H_2_SO_4_ at scan rate 2 mVs^-1^ before and after reductive-activation.


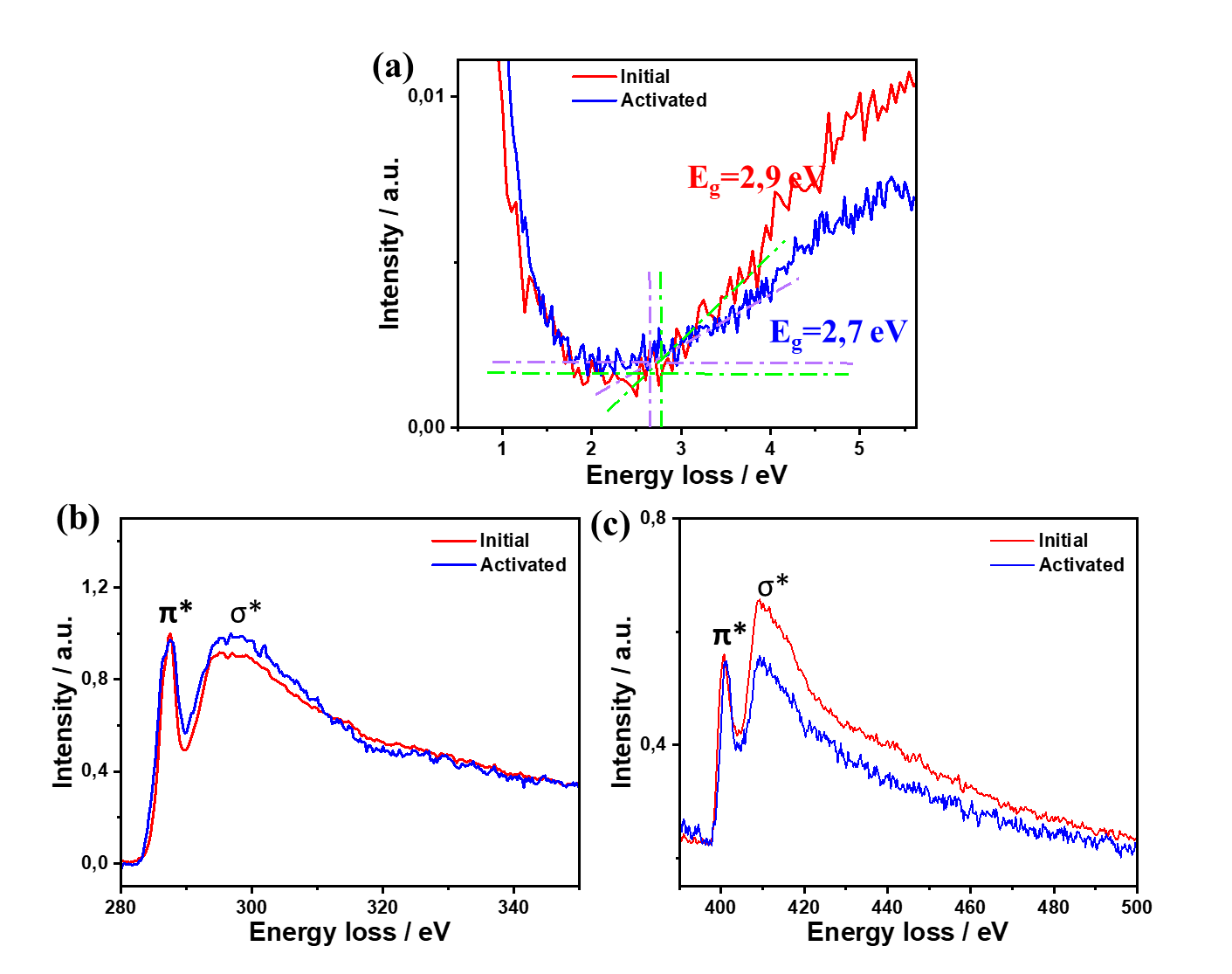


**Supplementary Figure 13** Reflection EELS spectra (a), EELS spectra of carbon (b) and nitrogen (c) of MnCo/g-C_3_N_4_ before and after activation.

**References**

1. B. Salah *et al.*, Synergistically interactive MnFeM (M = Cu, Ti, and Co) sites doped porous g-C3N4 fiber-like nanostructures for an enhanced green hydrogen production. *Green Chemistry* **25**, 6032-6040 (2023).

2. A. Abdelgawad *et al.*, Template-free synthesis of M/g-C3N4 (M= Cu, Mn, and Fe) porous one-dimensional nanostructures for green hydrogen production. *Journal of Electroanalytical Chemistry* **938**, 117426 (2023).

3. Z.-F. Huang *et al.*, Hollow cobalt-based bimetallic sulfide polyhedra for efficient all-pH-value electrochemical and photocatalytic hydrogen evolution. *Journal of the American Chemical Society* **138**, 1359-1365 (2016).

4. K. Wu *et al.*, Toward bifunctional overall water splitting electrocatalyst: general preparation of transition metal phosphide nanoparticles decorated N-doped porous carbon spheres. *ACS applied materials & interfaces* **10**, 44201-44208 (2018).

5. G. Li *et al.*, Cobalt–Cobalt Phosphide Nanoparticles@ Nitrogen‐Phosphorus Doped Carbon/Graphene Derived from Cobalt Ions Adsorbed Saccharomycete Yeasts as an Efficient, Stable, and Large‐Current‐Density Electrode for Hydrogen Evolution Reactions. *Advanced Functional Materials* **28**, 1801332 (2018).

6. S. A. Shah *et al.*, Nickel@ Nitrogen‐Doped Carbon@ MoS2 Nanosheets: An Efficient Electrocatalyst for Hydrogen Evolution Reaction. *Small* **15**, 1804545 (2019).

7. Y. Hu *et al.*, Ultrafine CoPS nanoparticles encapsulated in N, P, and S tri-doped porous carbon as an efficient bifunctional water splitting electrocatalyst in both acid and alkaline solutions. *Journal of Materials Chemistry A* **6**, 10433-10440 (2018).

8. X. Y. Yu *et al.*, Formation of Ni–Co–MoS2 nanoboxes with enhanced electrocatalytic activity for hydrogen evolution. *Advanced Materials* **28**, 9006-9011 (2016).

9. F. Zheng, H. Xia, S. Xu, R. Wang, Y. Zhang, Facile synthesis of MOF-derived ultrafine Co nanocrystals embedded in a nitrogen-doped carbon matrix for the hydrogen evolution reaction. *RSC advances* **6**, 71767-71772 (2016).

10. W. Zhou *et al.*, CoSe2 nanoparticles embedded defective carbon nanotubes derived from MOFs as efficient electrocatalyst for hydrogen evolution reaction. *Nano Energy* **28**, 143-150 (2016).

11. S. Mandegarzad, J. B. Raoof, S. R. Hosseini, R. Ojani, MOF-derived Cu-Pd/nanoporous carbon composite as an efficient catalyst for hydrogen evolution reaction: A comparison between hydrothermal and electrochemical synthesis. *Applied Surface Science* **436**, 451-459 (2018).

12. Y. Xie *et al.*, MOFs derived carbon nanotubes coated CoNi alloy nanocomposites with N-doped rich-defect and abundant cavity structure as efficient trifunctional electrocatalyst. *Applied Surface Science* **536**, 147786 (2021).

13. R. Nivetha, A. N. Grace, Manganese and zinc ferrite based graphene nanocomposites for electrochemical hydrogen evolution reaction. *Journal of Alloys and Compounds* **796**, 185-195 (2019).

14. B. Salah *et al.*, Synergistically interactive MnFeM (M= Cu, Ti, and Co) sites doped porous gC 3 N 4 fiber-like nanostructures for an enhanced green hydrogen production. *Green Chemistry* **25**, 6032-6040 (2023).

15. Y. Zheng *et al.*, Hydrogen evolution by a metal-free electrocatalyst. *Nature communications* **5**, 3783 (2014).

16. Y. Zhao *et al.*, Graphitic carbon nitride nanoribbons: graphene‐assisted formation and synergic function for highly efficient hydrogen evolution. *Angewandte Chemie International Edition* **53**, 13934-13939 (2014).

17. S. Shinde, A. Sami, J.-H. Lee, Electrocatalytic hydrogen evolution using graphitic carbon nitride coupled with nanoporous graphene co-doped by S and Se. *Journal of Materials Chemistry A* **3**, 12810-12819 (2015).

18. X. Zou, R. Silva, A. Goswami, T. Asefa, Cu-doped carbon nitride: Bio-inspired synthesis of H2-evolving electrocatalysts using graphitic carbon nitride (g-C3N4) as a host material. *Applied surface science* **357**, 221-228 (2015).

19. M. Chhetri, S. Maitra, H. Chakraborty, U. V. Waghmare, C. Rao, Superior performance of borocarbonitrides, B x C y N z, as stable, low-cost metal-free electrocatalysts for the hydrogen evolution reaction. *Energy & Environmental Science* **9**, 95-101 (2016).

20. J. Wen *et al.*, Fabricating the robust g-C3N4 nanosheets/carbons/NiS multiple heterojunctions for enhanced photocatalytic H2 generation: an insight into the trifunctional roles of nanocarbons. *ACS Sustainable Chemistry & Engineering* **5**, 2224-2236 (2017).

21. L. Yang, X. Wang, J. Wang, G. Cui, D. Liu, Graphite carbon nitride/boron-doped graphene hybrid for efficient hydrogen generation reaction. *Nanotechnology* **29**, 345705 (2018).

22. A. Durairaj, T. Sakthivel, S. Ramanathan, S. Vasanthkumar, Quenching-induced structural distortion of graphitic carbon nitride nanostructures: enhanced photocatalytic activity and electrochemical hydrogen production. *ACS omega* **4**, 6476-6485 (2019).

23. M. Gao *et al.*, Modification of layered graphitic carbon nitride by nitrogen plasma for improved electrocatalytic hydrogen evolution. *Nanomaterials* **9**, 568 (2019).

24. H. Zhao *et al.*, Preparation and electrocatalytic hydrogen evolution properties of nonmetallic functionalized carbon nanofiber catalysts. *Materials Research Express* **6**, 115509 (2019).

25. M. B. Idris, S. Devaraj, Mesoporous graphitic carbon nitride synthesized using biotemplate as a high-performance electrode material for supercapacitor and electrocatalyst for hydrogen evolution reaction in acidic medium. *Journal of Energy Storage* **26**, 101032 (2019).

26. L. Luo *et al.*, In situ construction of hierarchical graphitic carbon nitride homojunction as robust bifunctional photoelectrocatalyst for overall water splitting. *Journal of Chemical Technology & Biotechnology* **95**, 758-769 (2020).

27. S. Riyajuddin, S. Tarik Aziz, S. Kumar, G. D. Nessim, K. Ghosh, 3D‐Graphene Decorated with g‐C3N4/Cu3P composite: A Noble Metal‐free Bifunctional Electrocatalyst for overall water splitting. *ChemCatChem* **12**, 1394-1402 (2020).

28. Z. Jiang, C. Jia, B. Wang, P. Yang, G. Gao, Hexagonal g-C3N4 nanotubes with Pt decorated surface towards enhanced photo-and electro-chemistry performance. *Journal of Alloys and Compounds* **826**, 154145 (2020).

29. M. Mary Xavier *et al.*, Exploring The Effect of Precursors of Polymeric Carbon Nitride Nanosheets on their Photo and Electrocatalytic Applications. *ChemistrySelect* **5**, 12679-12689 (2020).

30. M. A. Ahsan *et al.*, Tuning the intermolecular electron transfer of low-dimensional and metal-free BCN/C60 electrocatalysts via interfacial defects for efficient hydrogen and oxygen electrochemistry. *Journal of the American Chemical Society* **143**, 1203-1215 (2021).

31. S. Kumar *et al.*, Strategy to improve the super-capacitive and hydrogen evolution performance of graphitic carbon nitrides via enrichment of carbon content. *Journal of Alloys and Compounds* **858**, 157671 (2021).

32. F. Nichols *et al.*, Platinum-complexed phosphorous-doped carbon nitride for electrocatalytic hydrogen evolution. *Journal of Materials Chemistry A* **10**, 5962-5970 (2022).

33. Z. Yu *et al.*, Single-atom Ir and Ru anchored on graphitic carbon nitride for efficient and stable electrocatalytic/photocatalytic hydrogen evolution. *Applied Catalysis B: Environmental* **310**, 121318 (2022).

34. H. He *et al.*, Constructing 3D interweaved MXene/graphitic carbon nitride nanosheets/graphene nanoarchitectures for promoted electrocatalytic hydrogen evolution. *Journal of Energy Chemistry* **67**, 483-491 (2022).

35. H. Choi *et al.*, Enhanced electrocatalytic full water-splitting reaction by interfacial electric field in 2D/2D heterojunction. *Chemical Engineering Journal* **450**, 137789 (2022).

36. E.-J. Lin *et al.*, Graphitic carbon nitride embedded with single-atom Pt for photo-enhanced electrocatalytic hydrogen evolution reaction. *Applied Surface Science* **615**, 156372 (2023).

37. M. Z. Rahman, J. Zhang, Y. Tang, K. Davey, S.-Z. Qiao, Graphene oxide coupled carbon nitride homo-heterojunction photocatalyst for enhanced hydrogen production. *Materials Chemistry Frontiers* **1**, 562-571 (2017).

38. J. Duan, S. Chen, M. Jaroniec, S. Z. Qiao, Porous C3N4 nanolayers@ N-graphene films as catalyst electrodes for highly efficient hydrogen evolution. *ACS nano* **9**, 931-940 (2015).

39. J. Zhang *et al.*, N, P‐codoped carbon networks as efficient metal‐free bifunctional catalysts for oxygen reduction and hydrogen evolution reactions. *Angewandte Chemie* **128**, 2270-2274 (2016).

40. C. Hu, L. Dai, Multifunctional carbon‐based metal‐free electrocatalysts for simultaneous oxygen reduction, oxygen evolution, and hydrogen evolution. *Advanced Materials* **29**, 1604942 (2017).

41. Q. Han *et al.*, Mesh‐on‐mesh graphitic‐C3N4@ graphene for highly efficient hydrogen evolution. *Advanced Functional Materials* **27**, 1606352 (2017).

42. Z. Pei *et al.*, Toward enhanced activity of a graphitic carbon nitride-based electrocatalyst in oxygen reduction and hydrogen evolution reactions via atomic sulfur doping. *Journal of Materials Chemistry A* **4**, 12205-12211 (2016).

43. M. B. Idris, T. Subramaniam, D. Sappani, Tailoring the electrocatalytic activity of mesoporous graphitic carbon nitride towards hydrogen evolution reaction by incorporation of amorphous carbon. *Diamond and Related Materials* **129**, 109359 (2022).

44. B. Zhang *et al.*, Self‐Adaptive Electronic Structure of Amphoteric Conjugated Ligand‐Modified 3 d Metal− C3N4 Smart Electrocatalyst by pH Self‐Response Realizing Electrocatalytic Self‐Adjustment. *ChemSusChem*, e202300078 (2023).

45. S. Y. Chong *et al.*, Tuning of gallery heights in a crystalline 2D carbon nitride network. *Journal of Materials Chemistry A* **1**, 1102-1107 (2013).

46. G. Algara‐Siller *et al.*, Triazine‐based graphitic carbon nitride: a two‐dimensional semiconductor. *Angewandte Chemie International Edition* **53**, 7450-7455 (2014).

47. F. K. Kessler *et al.*, Functional carbon nitride materials — design strategies for electrochemical devices. *Nature Reviews Materials* **2**, 17030 (2017).

48. S. Ren *et al.*, Porous, Fluorescent, Covalent Triazine-Based Frameworks Via Room-Temperature and Microwave-Assisted Synthesis. *Advanced Materials* **24**, 2357-2361 (2012).

49. N. Wang *et al.*, Design of palladium-doped g-C3N4 for enhanced photocatalytic activity toward hydrogen evolution reaction. *ACS Applied Energy Materials* **1**, 2866-2873 (2018).

50. H. Wang *et al.*, Fe‐Doped Porous g‐C3N4: An Efficient Electrocatalyst with Fe‐N Active Sites for Electrocatalytic Hydrogen Evolution Reaction under Alkaline Conditions. *ChemistrySelect* **7**, e202200306 (2022).

51. K. Eid *et al.*, Hierarchical Porous Carbon Nitride-Crumpled Nanosheet-Embedded Copper Single Atoms: An Efficient Catalyst for Carbon Monoxide Oxidation. *ACS Applied Materials & Interfaces* **14**, 40749-40760 (2022).

52. M. H. Vu, C. C. Nguyen, T. O. Do, Graphitic Carbon Nitride (g‐C3N4) Nanosheets as a Multipurpose Material for Detection of Amines and Solar‐Driven Hydrogen Production. *ChemPhotoChem* **5**, 466-475 (2021).

53. F. Qiao, J. Wang, S. Ai, L. Li, As a new peroxidase mimetics: The synthesis of selenium doped graphitic carbon nitride nanosheets and applications on colorimetric detection of H2O2 and xanthine. *Sensors and Actuators B: Chemical* **216**, 418-427 (2015).

54. Y. Li *et al.*, Bimetallic MnCo alloy nanoparticles decorated boron-doped carbon nanotubes as an active and durable electrode for supercapacitor. *Microporous and Mesoporous Materials* **309**, 110535 (2020).
